# An improved sample selection framework for learning with noisy labels

**DOI:** 10.1371/journal.pone.0309841

**Published:** 2024-12-05

**Authors:** Qian Zhang, Yi Zhu, Ming Yang, Ge Jin, Yingwen Zhu, Yanjun Lu, Yu Zou, Qiu Chen

**Affiliations:** 1 School of Information Technology, Jiangsu Open University, Nanjing, Jiangsu, China; 2 School of Computer and Electronic Information, Nanjing Normal University, Nanjing, Jiangsu, China; 3 School of Artificial Intelligence (School of Future Technology), Nanjing University of Information Science & Technology, Nanjing, Jiangsu, China; 4 Department of Electrical Engineering and Electronics, Graduate School of Engineering, Kogakuin University, Tokyo, Japan; Virtual University of Pakistan, PAKISTAN

## Abstract

Deep neural networks have powerful memory capabilities, yet they frequently suffer from overfitting to noisy labels, leading to a decline in classification and generalization performance. To address this issue, sample selection methods that filter out potentially clean labels have been proposed. However, there is a significant gap in size between the filtered, possibly clean subset and the unlabeled subset, which becomes particularly pronounced at high-noise rates. Consequently, this results in underutilizing label-free samples in sample selection methods, leaving room for performance improvement. This study introduces an enhanced sample selection framework with an oversampling strategy (SOS) to overcome this limitation. This framework leverages the valuable information contained in label-free instances to enhance model performance by combining an SOS with state-of-the-art sample selection methods. We validate the effectiveness of SOS through extensive experiments conducted on both synthetic noisy datasets and real-world datasets such as CIFAR, WebVision, and Clothing1M. The source code for SOS will be made available at https://github.com/LanXiaoPang613/SOS.

## Introduction

Deep neural networks (DNNs) are widely adopted for various vision tasks such as object detection [1], course recommendation [2], segmentation [3], image captioning [4], image denoising [5], corporate relative valuation [6], because of their excellent learning capabilities. However, DNNs require high-quality annotated training data, which can be costly to collect and contain data redundancy [7]. Image data sourced from the internet often requires manual annotation through crowdsourcing platforms, a process that consumes a significant amount of time and resources, making it less efficient. Furthermore, the crowdsourcing mechanism, which involves cross-annotating and voting, introduces instances with inaccurate annotations, known as noisy labels. Unfortunately, the robust memory capabilities of DNNs can lead to model overfitting noisy labels, resulting in reduced accuracy and discrimination. To address this challenge, one approach is to manually label a portion of the samples and treat the remaining samples as unlabeled data, processing them using semi-supervised techniques [8] and data quality assessment methods [9, 10] to improve performance. Another approach is to enhance model robustness through learning with noisy labels (LNL) methods. Early research in LNL, including techniques such as loss adjustment [11, 12], noise transition matrix estimation using robust architecture [13–17], label correction based on DNN predictions [18–24], robust loss functions [25–28], and sample selection [29–41], has shown promising results.

Loss adjustment involves modifying the loss of all training samples before backward propagation in DNNs to mitigate the influence of samples with noisy labels. Some approaches focus on designing robust architectures to model the noise transition matrix *T* ∈ [0,1]^*c×c*^, which reflects the probability of one category being mislabeled to another category, i.e., *T*_*ij*_: = *p* (*y^η^* = j|*y* = *i*), where the *c* is the category number, *y^η^* = *j* is the noisy label of a sample belonging to the *j*-th class and *y* = *i* is corresponding ground-truth label belonging to *i*-th class. Researchers have also explored using DNNs to correct noisy labels, leveraging the predictive capabilities of these models. This technique is called label correction. 42 has demonstrated that using a suitably modified loss function enables models trained on noisy datasets to achieve optimal Bayes risk, similar to their performance on clean datasets. Consequently, research on robust loss functions focuses on designing functions that enable models to effectively learn from clean labels while avoiding overfitting to noisy samples. Among numerous research avenues, sample selection methods have garnered widespread attention due to their state-of-the-art (SOTA) performance and ability to purify noisy datasets. As such, they have become mainstream in current LNL research. As the term suggests, sample selection aims to detect noisy labels and then filter out a subset of potentially clean labels for training. Early sample selection methods used dedicated architectures or training strategies to identify and remove potentially noisy samples. Although these methods achieved advanced performance, they left the information within noisy samples unutilized. Consequently, current SOTA sample selection methods incorporate semi-supervised learning (SSL) techniques for robust training, treating noisy samples as unlabeled data. However, these methods exhibit a significant gap between the size of the filtered, potentially clean subset, and the remaining unlabeled subset, particularly at high-noise levels. This gap, as shown in the experimental results in the Experimental results on synthetic datasets section, means that the label-free samples are not fully exploited, indicating potential for performance enhancement. To address this, we propose an improved sample **s**election framework with an **o**versampling **s**trategy (SOS). Inspired by UNCION [35], LongReMix [41], and SFA [42], SOS is a simple yet efficient method that mines useful information in label-free instances by combining an oversampling strategy with robust SSL techniques. This enhancement further boosts model performance. As evidenced by the experimental results in the Experimental results on synthetic datasets section, SOS maintains stable performance under high-noise conditions, demonstrating exceptional robustness against noisy labels. We applied SOS to several benchmark datasets, as in previous sample selection methods, and extensive experimental comparisons validate the effectiveness of our approach. Our main contributions are as follows:

1. We introduce a straightforward but effective sample selection framework with an oversampling strategy, which further utilizes information in label-free samples to achieve SOTA performance.
2. We propose a uniform sample selection approach, diverging from methods that predominantly rely on estimated noisy posterior probability, to enhance the robustness of DNNs and improve performance.
3. We introduce an oversampling strategy that complements SOTA one-stage sample selection methods for dataset division and robust training, setting our approach apart from two-stage methods and long-tailed learning research.
4. SOS exhibits more stable performance than current methods under high-noise levels, with a faster convergence rate.
5. Through extensive experiments across various noise types and rates, we demonstrate the superiority of SOS over existing SOTA methods, particularly under high-noise conditions.

The rest of this study is organized as follows: The Related works section reviews relevant studies on LNL. The Methodology sectiondetails the proposed SOS framework. The Experiments section discusses experimental results, and the conclusion section presents the conclusions.

## Related works

LNL research has emerged as a prominent area of study. The main research directions in this field can be categorized into five groups, as summarized in [43], including loss adjustment, noise transition matrix estimation using robust architecture, label correction based on DNN predictions, robust loss functions, and sample selection. Our work intersects with research on sample selection and long-tailed distribution.

### Research on sample selection for LNL

Sample selection methods have garnered significant attention for their SOTA performance. In general, these methods fall into two categories: those utilizing supervised techniques and those employing semi-supervised or unsupervised learning techniques. Research [12, 44] has empirically and theoretically shown that DNNs tend to fit clean samples initially but subsequently overfit noisy samples, resulting in lower losses for clean samples in early epochs. Therefore, early sample selection methods developed dedicated architectures or training strategies to identify and eliminate noisy labels based on this phenomenon. This entire process is supervised, involving only samples with potentially clean labels. For instance, Han et al. [29] introduced the co-teaching strategy using two duplicate networks to select clean samples based on the small loss criterion. Wei et al. [33] proposed the JoCoR framework, an evolution of the co-teaching strategy, where a sample is selected only if both networks predict the same category. Xia et al. [32] combined two multilayer perceptrons (MLPs) with co-teaching to identify clean samples for cross-training the networks.

More recent SOTA methods still filter samples with noisy labels based on loss, but instead of discarding them, they remove the labels and treat these samples as an unlabeled subset. This approach results in a labeled subset and an unlabeled subset, enabling semi-supervised or unsupervised learning techniques for training. Li et al. [34], for example, were the first to incorporate existing SSL techniques into sample selection for LNL, using a GMM to split the data and applying methods such as MixMatch [45] or FixMatch [46] for robust training. Following this, Ortego et al. [31] integrated contrastive learning (CL) to learn robust representations and categorize training samples into label-free and labeled sets. Karim et al. [35] combined Jensen-Shannon divergence (JSD) with unsupervised CL to facilitate robust training under noisy labels. In contrast, Li et al. [37] employed supervised CL to conduct sample selection and robust training. Similarly, Yao et al. [38] utilized JSD to estimate the samples’ likelihood of being clean or noisy, thereby categorizing training data and employing CL to enhance model robustness. Recently, Feng et al. [36] introduced the optimal transport theory to the sample selection process, yielding excellent performance. Diverging from previous methods that solely rely on loss [30, 34] to estimate the posterior probability of a sample containing a noisy label, Huang et al. [39] detected noisy samples by employing two GMMs to establish the relationship between representation and label-noisy annotations. Unlike earlier sample selection methods, which depend on a preset fixed threshold and are ineffective as epochs increase, Li et al. [40] propose a dynamic instance-specific selection method for LNL. Contrasting with previous sample selection methods that incorporate SSL techniques within the same training process for sample selection and robustness training, Cordeiro et al. [41] split these two optimization objectives into two processes.

### Research on long-tailed learning

Existing long-tailed learning (LTL) methods primarily address training datasets characterized by well-annotated yet imbalanced class distributions. In such datasets, some classes possess numerous samples, while others have significantly fewer. Most LTL methods, including those referenced in [44, 47–49], employ random oversampling and under-oversampling strategies to re-balance class representation during training. In LNL, the clean subset, being smaller than the label-free subset, presents a long-tailed distribution challenge, an area that has received limited research attention. To our knowledge, LongReMix [41] is the pioneer in integrating an SOS into LNL. However, it is a two-stage method that does not fully capitalize on the information available from label-free samples.

## Methodology

If all labels are well-annotated for a dataset with c-class, it can be classified as a clean dataset $D={\left\{(x_{i},y_{i})\right\}}_{i=1}^{n}$, where *x*_*i*_ ∈ ℜ*^ρ^* is the *i*-th input image, and *y*_*i*_∈{0,1}^*c*^ denotes its corresponding one-hot target. In this study, we employ two networks for training, each comprising an extractor *g*(⋅), a classifier *f*(⋅), and a projection header *h*(⋅) similar to simCLR [52]. The extracted feature by the extractor for *x*_*i*_ ∈ ℜ*^ρ^* is *g*(*x*_*i*_) and the representation via the projection header is denoted as *h*_*i*_ = *h*(*g*(*x*_*i*_)). The DNN prediction for *x*_*i*_ is denoted as *f*(*g*(*x*_*i*_)), simplified as *f*(*x*_*i*_) for simplicity. Typically, classification training methods predominantly utilize the Cross-Entropy (CE) loss function to train the DNNs. The optimization objective can be expressed as follows:

$$\underset{\Theta }{\min}L_{CE}\left(\Theta \right)=-\frac{1}{n}\sum_{i=1}^{n}y_{i}\log\left(f\left(x_{i}\right)\right).$$ (1)

**Fig 1:**
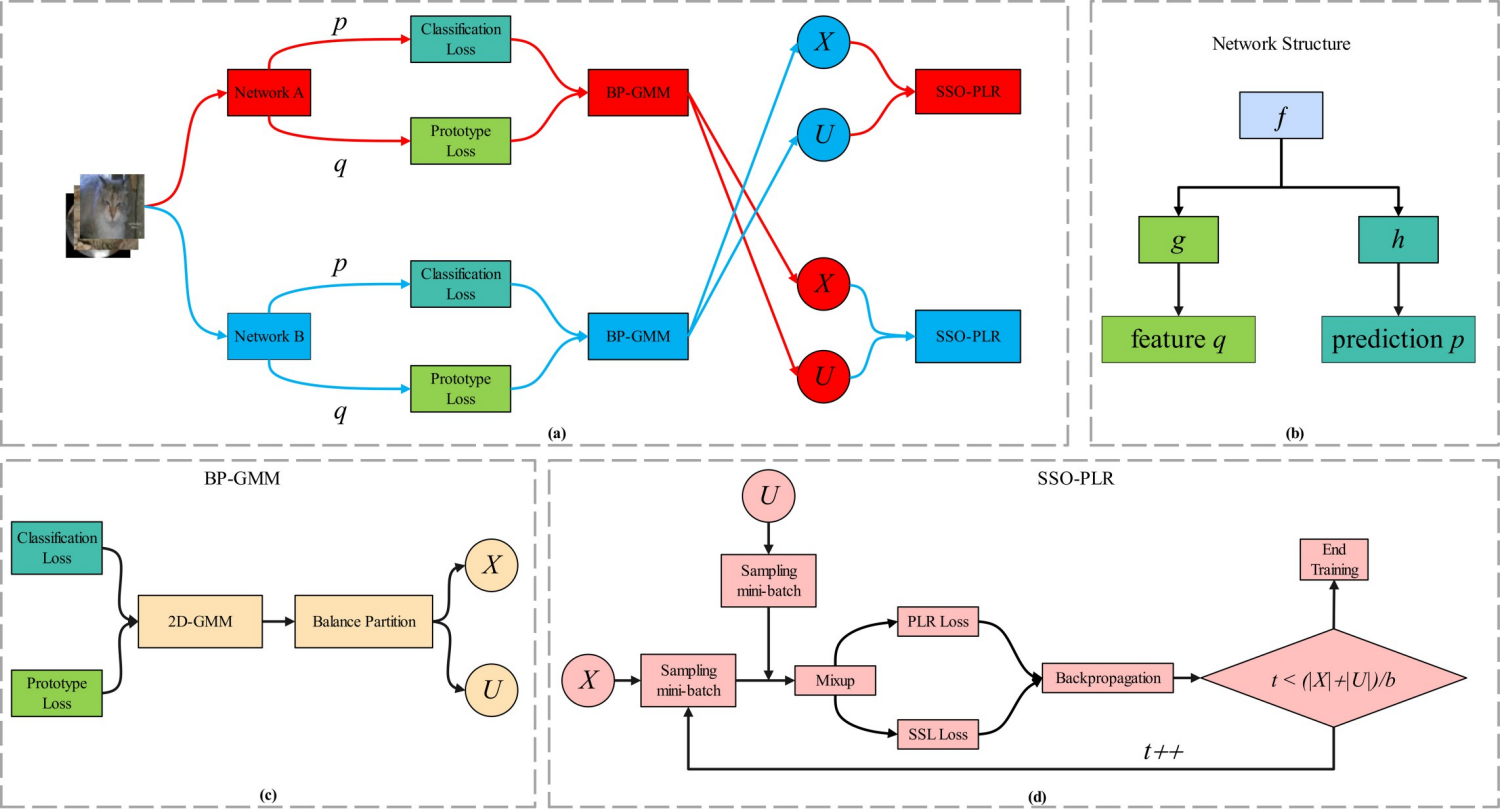
Overall framework of the SOS. Each network consists of an extractor *g*(⋅), a classifier *f*(⋅), and a projection header *h*(⋅), similar to simCLR [52]. During training, the dataset is divided into a labeled subset and an unlabeled subset through uniform selection, and then the useful information in the label-free samples is extracted by combining the oversampling strategy with a robust training process, where the robust training is performed using the SSL training technique MixMatch and unsupervised CL. In each network, the two subsets, derived separately from the two networks, are utilized for training. The training process is cyclic, involving repeated iterations of these steps.

Considering the gradient of Eq (1) and the powerful fitting capability of DNNs, it is clear that deep neural network models trained with CE loss attempt to fit all labels to the greatest extent possible. However, when the observed target *y*_*i*_ in the dataset contains inaccurate annotations (noisy labels $y_{i}^{\eta }$), with the increase of iteration, the DNNs, aided by the CE loss, will also fit these samples with noisy labels, leading to a significant decrease in classification performance and generalization ability [25, 50, 51]. This phenomenon is also known as the overfitting of DNNs to noisy labels in LNL fields [40]. Therefore, LNL aims to learn a global minimizer $f_{\eta }^{*}=\underset{f}{\arg\;\min}\left(R^{\eta }\left(f\right)=E_{(x,y^{\eta })∼D^{\eta }}\left[L\left(x\right),y^{\eta }\right]\right)$ on noisy datasets $D^{\eta }={\left\{(x_{i},y_{i}^{\eta })\right\}}_{i=1}^{n}$ which has the same probability of misclassification as that of f^*^ (a global minimizer of Eq (1) in the noise free dataset) [50], i.e., $f^{*}=f_{\eta }^{*}$. The comprehensive framework of the SOS is depicted in Fig 1, while Algorithm 2 provides an in-depth illustration of the process.

### Uniform selection approach

The SOS method combines an additional oversampling mechanism with the uniform sample selection approach and robust training. To elucidate, we first introduce the uniform selection approach. Traditional sample selection methods predominantly depend on the estimated noise posterior probability, derived using GMM to model the loss distribution. However, these methods do not ensure a uniform number of samples for each class in the clean set. Addressing this limitation, Karim et al. [35] propose the common uniform sample selection, which employs JSD instead of GMM for detecting noisy labels. As illustrated in Fig 1, two pre-trained networks are utilized to partition the training set. The predictions of these networks for the same input *x*_*i*_ via the softmax layer are denoted as $p_{i}^{1}=\text{softmax}\left(f_{1}\left(x_{i}\right)\right)=\left[p_{i1}^{1},\cdots ,p_{ic}^{1}\right]$ and $p_{i}^{2}=\text{softmax}\left(f_{2}\left(x_{i}\right)\right)=\left[p_{i1}^{2},\cdots ,p_{ic}^{2}\right]$, respectively, where $p_{ic}^{1}$is the *c*-th component of the prediction $p_{i}^{1}$ from network 1. JSD is also used to measure the disagreement between the predictions from the two networks and the observed one-hot label $y_{i}^{\eta }\in {\left\{0,1\right\}}^{c}$:

$$d_{i}=\frac{1}{2}\times \left(JSD\left(y_{i}^{\eta },p_{i}^{1}\right)+JSD\left(y_{i}^{\eta },p_{i}^{2}\right)\right)$$ (2)

where the JSD function is

$$JSD\left(y_{i}^{\eta },p_{i}\right)=\frac{1}{2}\times \left(KL\left(y_{i}^{\eta }\left\|\frac{y_{i}^{\eta }+p_{i}}{2}\right.\right)+KL\left(p_{i}\left\|\frac{y_{i}^{\eta }+p_{i}}{2}\right.\right)\right)$$ (3)

and *KL*(⋅‖⋅) is the Kullback-Leibler Divergence. Here,

$$KL\left(y_{i}^{\eta }\left\|\frac{y_{i}^{\eta }+p_{i}}{2}\right.\right)=\sum_{j=0}^{c-1}y_{ij}^{\eta }\;\log\left(\frac{2\times y_{ij}^{\eta }}{y_{ij}^{\eta }+p_{ij}}\right),$$ (4)

and $KL\left(p_{i}\left\|\frac{y_{i}^{\eta }+p_{i}}{2}\right.\right)$ can be calculated using a similar formula as Eq (4).

Assuming that the disagreements corresponding to *j*-th class after the sorting is $d^{\left(j\right)}=\mathrm{sort}\left(\left\{d_{i}\left|1\left(y_{ij}^{\eta }=1\right),\forall (x_{i},y_{i}^{\eta })\in D^{\eta }\right.\right\}\right)$, *j*∈{1,2, …,*c*}, the sample selection is based on the calculated disagreement for all training data. A sample is selected if its disagreement value falls within the first R portion (in ascending order) of all the disagreement values for the class indicated by its observed label. This can be mathematically expressed as follows:

$$D_{l}=\left\{(x_{i},y_{i}^{\eta })\left|d_{i}\in d^{\left(j\right)}\left[0:R\right],1\left(y_{ij}^{\eta }=1\right);\forall (x_{i},y_{i}^{\eta })\in D^{\eta },\forall j\in \left\{1,\cdots ,c\right\}\right.\right\},$$ (5)

**Fig 2:**
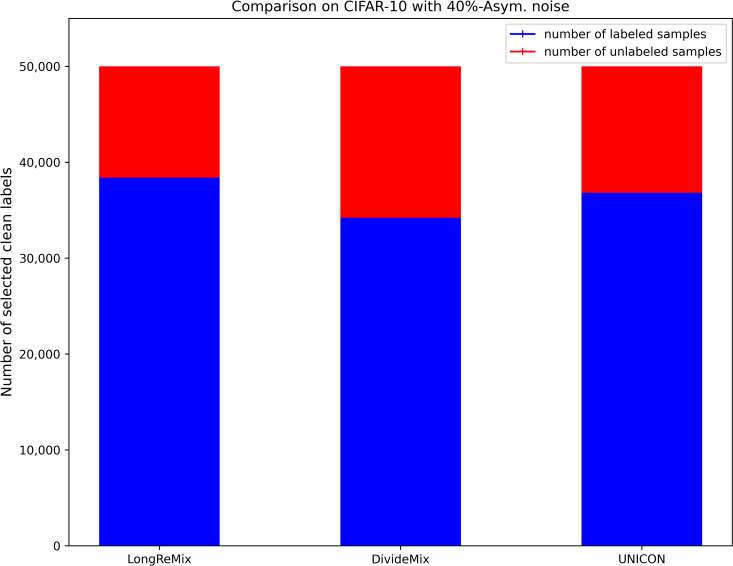
The proportions of labeled and unlabeled samples divided by existing SOTA methods on CIFAR datasets with 40%-asym.

**Fig 3:**
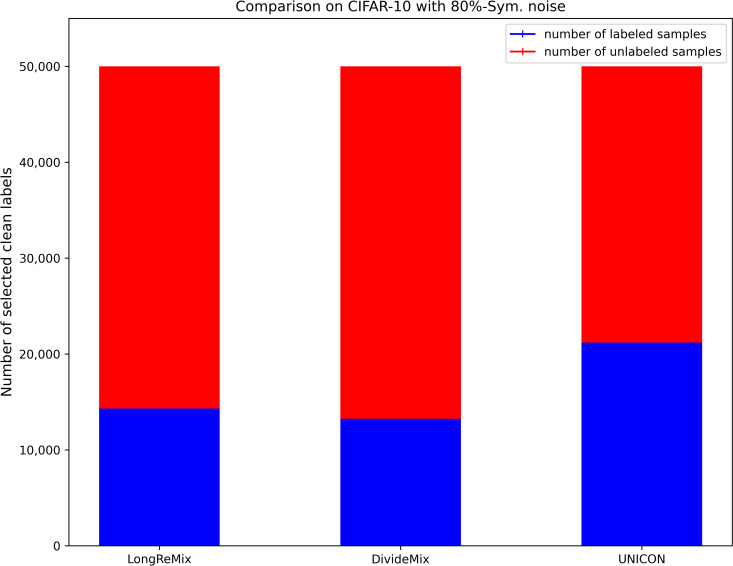
The proportions of labeled and unlabeled samples divided by existing SOTA methods on CIFAR datasets with 80%-sym.

**Fig 4:**
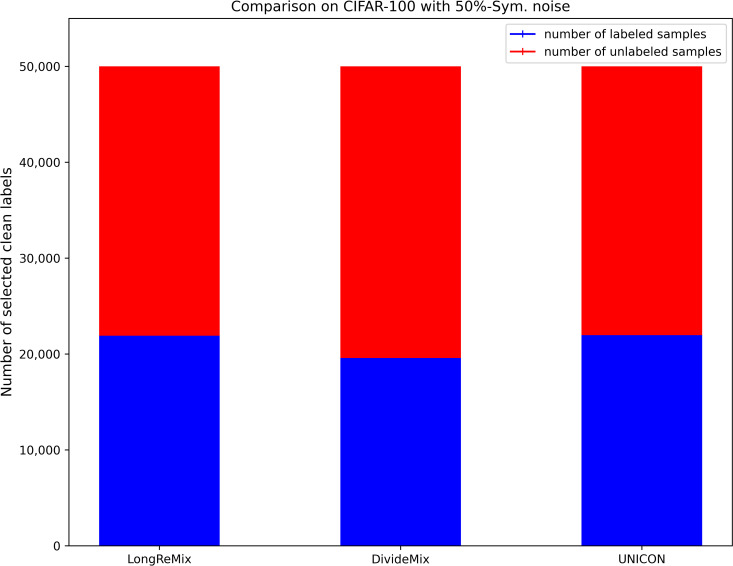
The proportions of labeled and unlabeled samples divided by existing SOTA methods on CIFAR datasets with 50%-sym.

**Fig 5:**
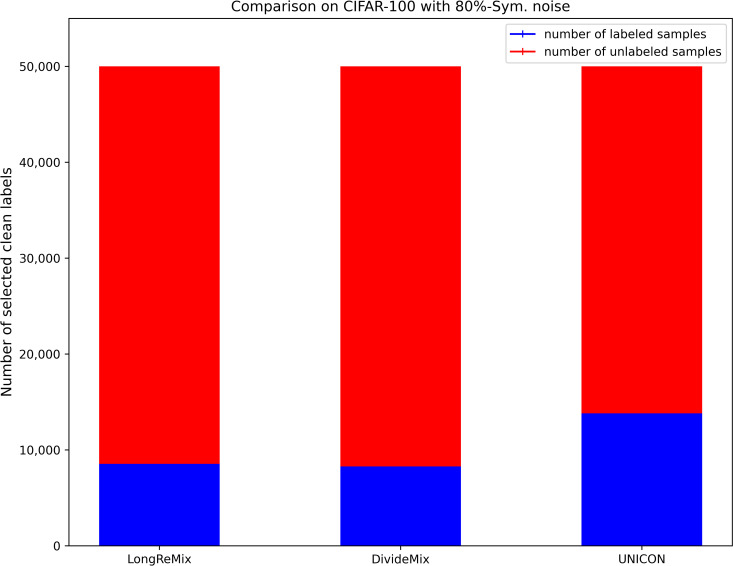
The proportions of labeled and unlabeled samples divided by existing SOTA methods on CIFAR datasets with 80%-sym.

R is calculated as follows:

$$R=\frac{c}{n}\times \sum_{i=1}^{n}1\left(d_{i}<d_{ts}\right),$$ (6)

where **1**(⋅) is an indicator function, and *d*_*ts*_ is determined by Eq (7):

$$d_{ts}=\left\{\begin{array}{c} d_{avg}-\left(d_{avg}-d_{\min}\right)/\tau ,\;\text{if}\;d_{avg}\geq d_{\mu }\\ d_{avg},\;\text{otherwise} \end{array}\right.$$ (7)

where *d*_*avg*_ and *d*_min_ are the average disagreement and minimum values overall training samples, respectively. *τ* and *d*_*μ*_ are the two hyperparameters used for transferring more samples to the unlabeled set, thus the size of the labeled set *D*_*l*_ (Eq (5)) is generally smaller than the size of the unlabeled set *D*_*ul*_ = {(*x*_*i*_)|*x*_*i*_∈*D^η^*/*D*_*l*_} under high-noise rates when using Eq (5). We illustrate the proportions of labeled and unlabeled samples divided by several SOTA sample selection methods (e.g.,) through a bar chart [54]. As shown in Figs 2–5, it is evident that in high noise rate scenarios (i.e., from 40%-asym. to 80%-sym. on CIFAR-10, and from 50%-sym. to 80%-sym. in CIFAR-100), the sizes of labeled subsets partitioned by these methods, including the baseline method (UNICON) in this paper, are significantly smaller than those of the unlabeled subsets.

### Robust SSL training with oversampling strategy

Most current SOTA sample selection methods employ SSL techniques to train DNNs simultaneously on labeled and unlabeled sets. They sample an equal number of labeled and unlabeled samples in each epoch, where the number is dependent on the size of the labeled subset. This strategy is effective for low-noise scenarios as the number of labeled samples is greater than that of unlabeled samples (as shown in Figs 2 and 4). However, as shown in Figs 3 and 5, under high-noise conditions, the size of the labeled set is often smaller than that of the unlabeled set. Consequently, this imbalance can interrupt robust SSL training due to the premature depletion of labeled samples in the data-loader. Such interruptions prevent many unlabeled samples from being learned by the DNNs, leaving room for performance enhancement.

To address this issue, we introduce an oversampling strategy. Commonly used in LTL, oversampling involves sampling more frequently from classes with fewer instances (rare classes) to maintain class balance. In this study, since |*D*_*l*_| ≪ |*D*_*ul*_|, we consider *D*_*l*_ as a rare class and *D*_*ul*_ as s a massive class. To prevent training from prematurely terminating due to the exhaustion of labeled samples in the data-loader, it is necessary to oversample more training data from the labeled set. This approach compels the DNNs to assimilate more useful information from the unlabeled samples, which the previous SSL training process might have overlooked. Consequently, the pseudocode for the robust SSL training incorporating the SOS, inspired by [41], is presented below.

**Algorithm 1** oversampling during robust SSL training
**Input:** the labeled set *D*_*l*_ and the unlabeled set *D*_*ul*_, two networks *f*_1_ and *f*_2_, batch-size *b*, robust SSL training function *F*_*ssl*_ (*B*_*labeled*_,*B*_*unlabeled*_,*f*_1_,*f*_2_);
Calculate the number of iterations in the labeled set based on the batch size *b*: ${\mathrm{iter}}_{\max}=\frac{\left(\left|D_{l}\right|+\left|D_{ul}\right|\right)}{b}+1$;
Initialize the current iteration of the labeled data-loader: iter_*cont*_ = 0;
**while** iter_*cont*_ < iter_max_:
 sample a mini-batch $B_{labeled}=\left\{\left(x_{i},y_{i}^{\eta }\right)\in D_{l};i\in \left\{1,\ldots ,b\right\}\right\}$ from *D*_*l*_;
 sample a mini-batch *B*_*ulabeled*_ = {(*x*_*i*_)∈*D*_*ul*_;*i* ∈{1, …,*b*}} from *D*_*ul*_;
 perform robust SSL training based on the current two mini batches: *F*_*ssl*_ (*B*_*labeled*_,*B*_*unlabeled*_,*f*_1_,*f*_2_);
 add the iteration of the labeled data-loader: iter_*cont*_ + = 1;
 **if** iter_*cont*_ ≥ iter_max_:
  break;
 **end if**
**end while**
**Output:** two networks *f*_1_ and *f*_2_.

Since these unlabeled samples have several potential representations useful for unsupervised CL, we adhere to the methodologies in [31, 35, 37] to integrate the CL method into the robust SSL training process post-division to further improve the robustness and performance of the model.

Below, we describe the robust SSL training method *F*_*ssl*_ (*B*_*labeled*_,*B*_*unlabeled*_,*f*_1_,*f*_2_) employed in this study. Consistent with the MixMatch for LNL approach used in previous works [34–40], for each sample *x_i_* ∈ *B*_*labeled*_ ∪*B*_*unlabeled*_, we initially perform two weak data augmentations i.e., *x*_*i*,1_,*x*_*i*,2_ = w_da(*x*_*i*_). Subsequently, label co-guessing is performed for both labeled and unlabeled samples, as depicted below:

$$\left\{\begin{array}{c} {\hat{y}}_{i}=d_{i}^{t}\cdot y_{i}^{\eta }+\left(1-d_{i}^{t}\right)\cdot \left(\frac{1}{2}\cdot \sum_{m=1}^{2}f_{cur}\left(x_{i,m}\right)\right),\;\forall \left(x_{i},y_{i}^{\eta }\right)\in B_{labeled}\\ {\hat{y}}_{i}=sharpen\left(\frac{1}{2}\cdot \sum_{m=1}^{2}\left(f_{1}\left(x_{i,m}\right)+f_{2}\left(x_{i,m}\right)\right)\right),\;\forall \left(x_{i}\right)\in B_{unlabeled} \end{array}\right.,$$ (8)

where the refined label ${\hat{y}}_{i}$ is derived by weighting the original observed targets $y_{i}^{\eta }$ and predictions from the two instances of **weak data augmentation** *x*_*i*,1_,*x*_*i*,2_ = *weak_aug*(*x*_*i*_) applied on the current training network *f*_*cur*_ when $\left(x_{i},y_{i}^{\eta }\right)$ belongs to *B*_*labeled*_. Otherwise, the guessed label ${\hat{y}}_{i}$ is determined solely by averaging the predictions from two networks *f*_1_ and *f*_2_, under the two instances of weak data augmentation wherein $\left(x_{i},y_{i}^{\eta }\right)$ belongs to *B*_*unlabeled*_, and *sharpen*(⋅) is expressed as follows:

$$sharpen\left({\bar{f}}_{i}\right)={\left({\bar{f}}_{ij}\right)}^{S}/\sum_{j=0}^{c-1}{\left({\bar{f}}_{ij}\right)}^{S}.$$ (9)

where *S* is the temperature parameter, ${\bar{f}}_{i}=\frac{1}{2}\cdot \sum_{m=1}^{2}\left(f_{1}\left(x_{i,m}\right)+f_{2}\left(x_{i,m}\right)\right)$ is the average prediction of two networks on two weak data augmentations of input *x*_*i*_, and ${\bar{f}}_{ij}$ represents the *j*-th component of ${\bar{f}}_{i}$. After the label co-guessing step, we initially replace the original label $y_{i}^{\eta }$ of sample *x*_*i*_ with the refined label ${\hat{y}}_{i}$. Subsequently, we combine ${\hat{y}}_{i}$ with two instances of **strong data augmentation** *x*_*i*,3_,*x*_*i*,4_ = *strong_aug*(*x*_*i*_) to form two new pairs $\left(x_{i,m},{\hat{y}}_{i}\right),m\in \left\{3,4\right\}$. Finally, two new mini batches are obtained:

$$\begin{array}{l} {B^{\prime }}_{labeled}=\left\{\left(x_{i,m},{\hat{y}}_{i}\right)\left|\left(x_{i},y_{i}^{\eta }\right)\in B_{labeled};m\in \left\{3,4\right\},i\in \left\{1,\ldots ,b\right\}\right.\right\}\\ {B^{\prime }}_{unlabeled}=\left\{\left(x_{i,m},{\hat{y}}_{i}\right)\left|\left(x_{i}\right)\in B_{unlabeled};m\in \left\{3,4\right\},i\in \left\{1,\ldots ,b\right\}\right.\right\} \end{array}.$$ (10)

Here *B*_*labeled*_ and *B*_*unlabeled*_ are the two mini batches sampled from *D*_*l*_ and *D*_*ul*_ respectively, as illustrated in Algorithm 1, and *b* is the batch size. Through Eq (10), each input *x*_*i*_ in *B*_*labeled*_ and *B*_*unlabeled*_ is augmented into two inputs *x*_*i*,3_ and *x*_*i*,4_ with strong data augmentation operation.

The loss function of the SSL training based on the refined labels at *t-*th epoch is expressed as follows:

$$L_{ssl}=-\frac{1}{N_{1}}\sum_{i=1}^{N_{1}}CE\left({\bar{y}}_{i},\;f\left({\bar{x}}_{i}\right)\right)+\frac{\lambda_{u}}{N_{2}}\sum_{i=1}^{N_{2}}{\left\|{\bar{y}}_{i}-f\left({\bar{x}}_{i}\right)\right\|}_{2}^{2}-\lambda_{reg}\cdot CE\left(\pi ,\;\frac{1}{N_{1}+N_{2}}\sum_{i=1}^{N_{1}+N_{2}}f\left({\bar{x}}_{i}\right)\right)$$ (11)

where *N*_1_ is the size of ${B^{\prime }}_{labeled}$, *N*_2_ is the size of ${B^{\prime }}_{unlabeled}$. $\pi ={\left[\frac{1}{c}\right]}^{c}$ represents a c-dimensional vector where each element is 1/*c* (i.e., to keep the model’s predictions uniformly distributed). CE is given in Eq (1), λ_*u*_ and *λ_reg_* are predefined tradeoffs. Furthermore, $\left({\bar{x}}_{i},{\bar{y}}_{i}\right)\in {B^{\prime \prime }}_{labeled}$ and $\left({\bar{x}}_{i},{\bar{y}}_{i}\right)\in {B^{\prime \prime }}_{unlabeled}$ are generated from ${B^{\prime }}_{labeled}$ and ${B^{\prime }}_{unlabeled}$ using the Mixup [53], as employed in previous studies. The process of generation is as follows:

$$\begin{array}{l} B_{labeled}^{"}=\left\{\left({\bar{x}}_{i},{\bar{y}}_{i},w_{i}^{t}\right)|{\bar{x}}_{i},{\bar{y}}_{i}=\lambda \cdot \left(x_{i},{\hat{y}}_{i}\right)+\left(1-\lambda \right)\cdot \left(x_{j},{\hat{y}}_{j}\right),\forall \left(x_{i},{\hat{y}}_{i}\right)\in B_{labeled}^{'},\forall \left(x_{j},{\hat{y}}_{j}\right)\in B_{labeled}^{'}\cup B_{unlabeled}^{'}\right\}\\ B_{unlabeled}^{"}=\left\{\left({\bar{x}}_{i},{\bar{y}}_{i},w_{i}^{t}\right)|{\bar{x}}_{i},{\bar{y}}_{i}=\lambda \cdot \left(x_{i},{\hat{y}}_{i}\right)+\left(1-\lambda \right)\cdot \left(x_{j},{\hat{y}}_{j}\right),\forall \left(x_{i},{\hat{y}}_{i}\right)\in B_{unlabeled}^{'},\forall \left(x_{j},{\hat{y}}_{j}\right)\in B_{labeled}^{'}\cup B_{unlabeled}^{'}\right\} \end{array}$$ (12)

where *λ* = *Beta*(*α*,*α*) and *α* is a predefined hyperparameter. Using the methodologies outlined in [31, 35, 37], we integrate the CL method into the robust SSL training process when the input is $x_{i}\in {B^{\prime }}_{unlabeled}$, yielding a total optimization objective expressed as follows:

$$L_{tot}=L_{ssl}-\frac{\lambda_{cl}}{2N_{2}}\sum_{x_{i}\in {B^{\prime }}_{unlabeled}}\left(CL\left(2i-1,2i\right)+CL\left(2i,2i-1\right)\right),$$ (13)

where ${B^{\prime }}_{unlabeled}$ is obtained from Eq (10), *λ_cl_* = 0.025 is a coefficient, and $CL\left(i,j\right)=-\log\frac{\exp\left(h_{i}\cdot h_{j}/0.05\right)}{\sum_{x_{k}\in {B^{\prime }}_{unlabeled},\;i\neq k}\exp\left(h_{i}\cdot h_{k}/0.05\right)}$. Ultimately, we employ Eq (13) and Algorithm 1 to determine the global minimizer $f_{\eta }^{*}$ during the robust SSL training.

### Proposed SOS framework

We propose the SOS framework, an enhancement of the uniform sample selection method discussed inthe Uniform selection approach section, integrated with the robust SSL training and SOS outlined in the Robust SSL training with oversampling strategy section. This one-stage method efficiently divides the training data and conducts robust training within the same epoch. An overview of our method is depicted in Fig 1, with the detailed workflow presented in Algorithm 2.

**Algorithm 2** SOS
**Input:** the training set *D^η^*, two networks *f*_1_, *f*_2_, the tradeoff factors *λ_u_*, *λ_reg_*, hyperparameter α, filtering factors *d_μ_* and *τ*, *T*_*w*_ is the warm-up epochs, *T*_*tot*_ is the total training epochs, temperature parameter *S*, batch-size *b*;
**for** *t* = 1 **to** *T*_*tot*_ **do:**
 **if** *t* < *T_w_*:
  pre-train two networks *f*_1_ and *f*_2_ based on the original training set *D^η^* using CE loss function;
 **else**:
  //the training of network *f*_1_;
  *f*_*cur*_ = *f*_1_;
  //divide the training data via the uniform sample selection approach as illustrated in the Uniform selection approach section;
  construct the labeled and unlabeled sets *D*_*l*_ and *D*_*ul*_ for network *f*_1_ using Eq (5);
  perform robust SSL training on network *f*_1_ employing the oversampling strategy, as outlined in Algorithm 1;
  //the training of network *f*_2_;
  *f*_*cur*_ = *f*_2_;
  //divide the training data via the uniform sample selection approach as illustrated in the Uniform selection approach section;
  construct the labeled and unlabeled sets *D*_*l*_ and *D*_*ul*_ for network *f*_2_ using Eq (5);
  perform robust SSL training on network *f*_2_ employing the oversampling strategy, as outlined in Algorithm 1;
 **end if**
 *t* = *t* +1  //incremental training epochs.
**end for**
**Output**: the labeled set *D*_*l*_, two robust networks *f*_1_ and *f*_2_.

## Experiments

In this section, we evaluate the performance of the SOS framework on both synthetic and real-world datasets with noise. The characteristics of the datasets used are described below. It is worth noting that the hyperparameters used in the experiments for each dataset in this paper are basically consistent with those used in UNICON and DivideMix. All experiments on the CIFAR datasets in this paper are conducted on a server running Windows 11, equipped with a single 4090 GPU and a 13900K CPU. Experiments on other datasets are conducted on a server running Windows Server 2016, equipped with a single A800 GPU and an Xeon 6248 CPU. The IDE used for all experiments is PyCharm 2023, and the model framework is PyTorch 1.8.0.

### The details of datasets

#### The details of CIFAR-10 and CIFAR-100

**Fig 6:**
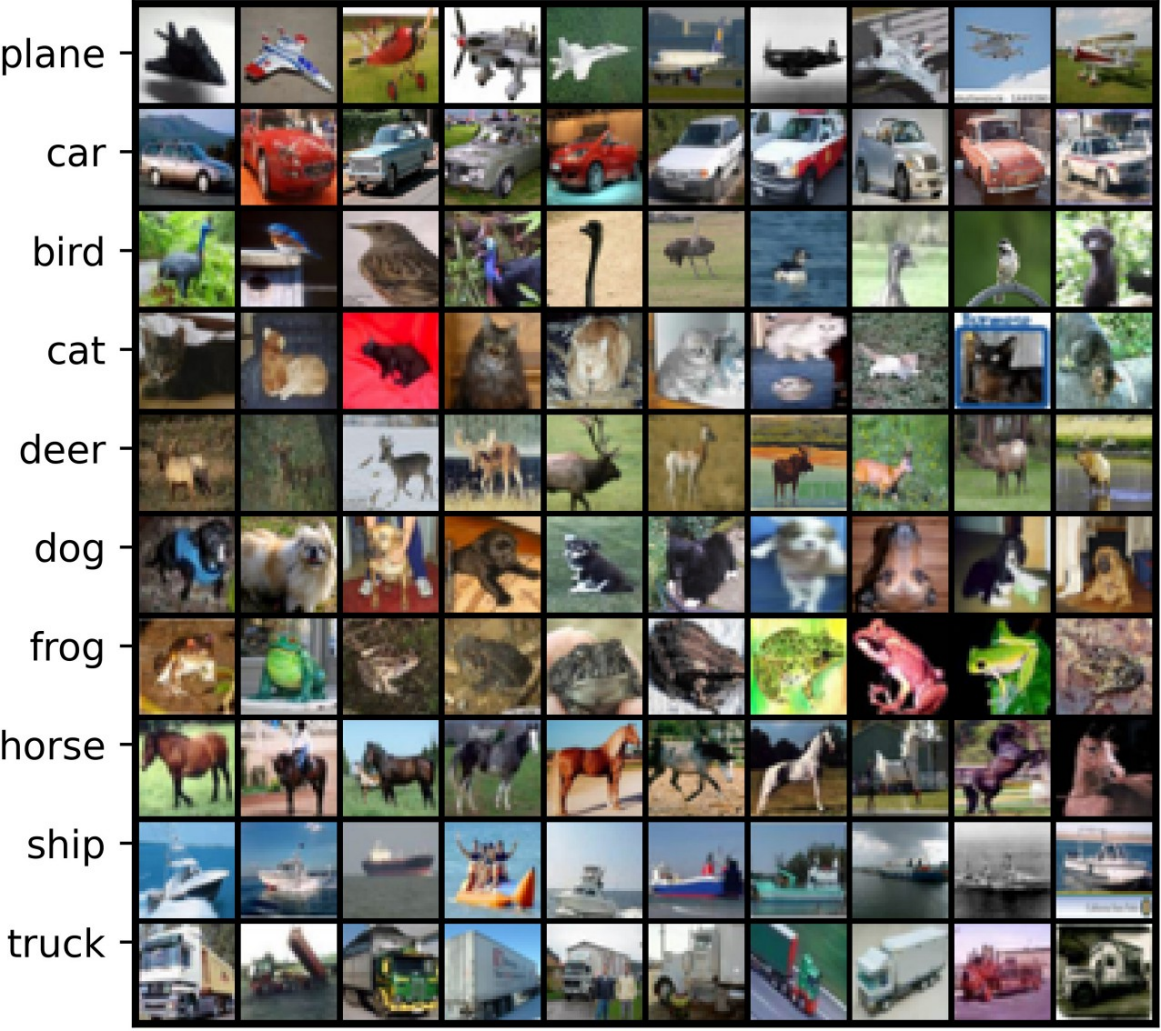
Visualizing samples from CIFAR-10.

**Fig 7:**
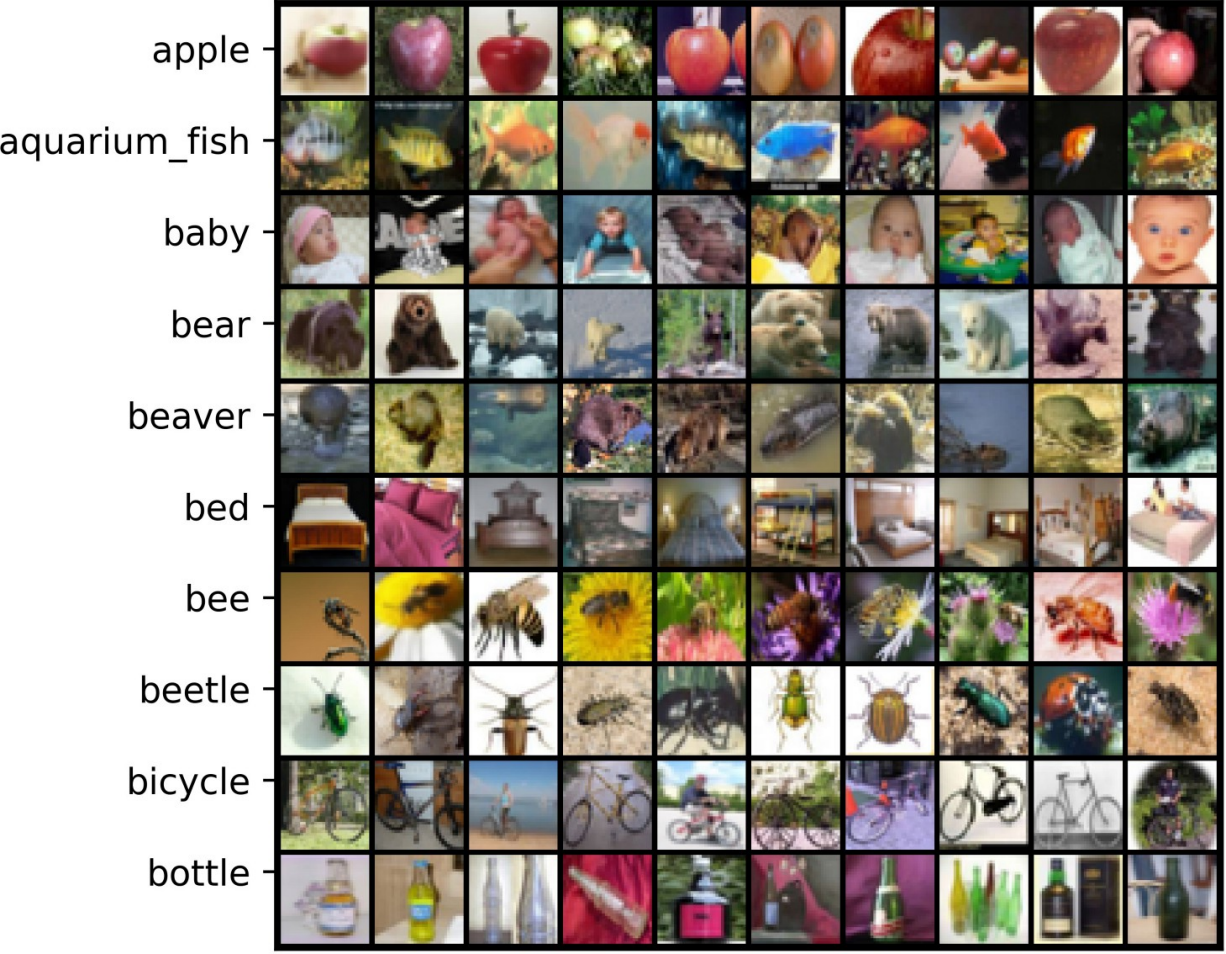
Visualizing samples from CIFAR-100.

*Basic overview*. CIFAR-10 and CIFAR-100 [55] are two clean dataset with 10 and 100 categories, respectively. Each dataset contains 50K training data and 10K testing data. Table 1 outlines the basic characteristics of them and Figs 6 and 7 display sample features of some classes in CIFAR-10 and CIFAR-100.

**Table 1:** Summary of datasets.

Datasets	Class	Training	Test	Original Size	Resize
CIFAR-10	10	50*K*	10*K*	32×32	32×32
CIFAR-100	100	50*K*	10*K*	32×32	32×32
CIFAR-N	10	50*K*	10*K*	32×32	32×32
WebVision	1000	2.4*M*	*50K*	320×320	229×229
Clothing1M	14	1*M*+48*K*	10*K*	256×256	224×224

*Synthesis of noisy labels*. Given the challenge in determining noise characteristics in real-world datasets, prior studies often utilize CIFAR-10/100 [55] to create controlled synthetic label noise at various rates, including both symmetric and asymmetric types, to test the efficacy of proposed methods. Table 1 summarizes these two datasets, noting that only the labels of the training data are altered with generated synthetic noisy labels. Symmetric noise implies that each sample’s target has a probability *η*/(*c*-1) of being randomly assigned into any category, and a 1-*η* chance of remaining unchanged. Asymmetric noise involves a fixed probability *η* of each target being mapped to a predetermined class. Notably, the asymmetric noise in CIFAR-10 mimics real-world label noise structure, exemplified by mappings, i.e., *truck* → *automabile*, *bird* → *airplane*, *deer* → *house*, *cat* ⇄ *dog*. In CIFAR-10, the asymmetric noise transition matrix is relatively sparse, while in CIFAR-100, asymmetric noisy labels are generated by shifting each target to the subsequent category of its superclass, resulting in a denser transition matrix.

*Experimental setup*. To evaluate the robustness of SOS on these datasets, we use PreAct ResNet-18, aligning with previous studies. The hyperparameters for CIFAR-10/100 are detailed in Table 2. We set *λ_u_* = 30 for all CIFAR-10 experiments, except for the 10%-asymmetric and 20% symmetric label noise (10%-asym and 20%-sym) scenario, where *λ_u_* = 0. Although using customized hyperparameter settings for certain noise scenarios could achieve better performance, to demonstrate the robustness of the hyperparameters of our method and to avoid additional ablation experiments, as well as to ensure fair comparison with previous methods like UNICON, DivideMix, we employ almost identical settings for all noise scenarios on this dataset. The hyperparameter settings are identical to UNICON. The learning rate undergoes linear reduction post-warm-up. The weak data augmentation (w_da) follows previous works’ protocols, such as mean subtraction and random flip, while the strong data augmentation (s_da) adopts the CIFAR10-Policy [59].

**Table 2:** Settings of the hyperparameters used in this study.

Dataset	CIFAR10	CIFAR100	CIFAR-10/100N	Clothing1M	WebVision
Backbone	PreAct ResNet-18		ResNet-34	ResNet-50	Inception-ResNet-v2
Learning rate	0.02	0.02	0.02	0.002	0.01
Optimizer	SGD				
Weight decay	0.0005			0.001	0.001
Momentum	0.9				
*b*	64		128	32	32
*T*_*w*_	10	30	10/30	15	
*T*_*tot*_	300			200	100
*d*_*μ*_	0.5			0.7	0.7
*λ_reg_*	1				
*S*	2				
*α*	4			0.5	0.5
τ	0.5				

#### The details of CIFAR-N

*Basic overview*. Although synthetic label noise can be generated as described above, modeling real-world noise patterns accurately remains challenging [56]. In addition, many existing real-world noisy datasets complicate the analysis of proposed LNL methods due to the absence of true labels and their large sizes. To address this, Wei et al. [56] developed CIFAR-N, a controllable, moderately sized real-world noisy dataset based on the training images from CIFAR-10 and CIFAR-100. CIFAR-N comprises five types of noise labels for CIFAR-10: aggregate (9.03%), random1-3 (17.23%/18.23%/17.64%), and worst (40.21%); one type for CIFAR-100, namely noisy (40.2%). Table 1 outlines the basic characteristics of CIFAR-N and the features of this dataset are shown in Figs 6 and 7.

*Experimental setup*. The details of the hyperparameters used are outlined in Table 2. The learning rate undergoes linear reduction after warm-up, and we set *λ_u_* = 0 for all noise types except for the worst and noisy types, where *λ_u_* = 30. The data augmentation strategy follows that of CIFAR-10/100.

#### The details of WebVision

**Fig 8:**
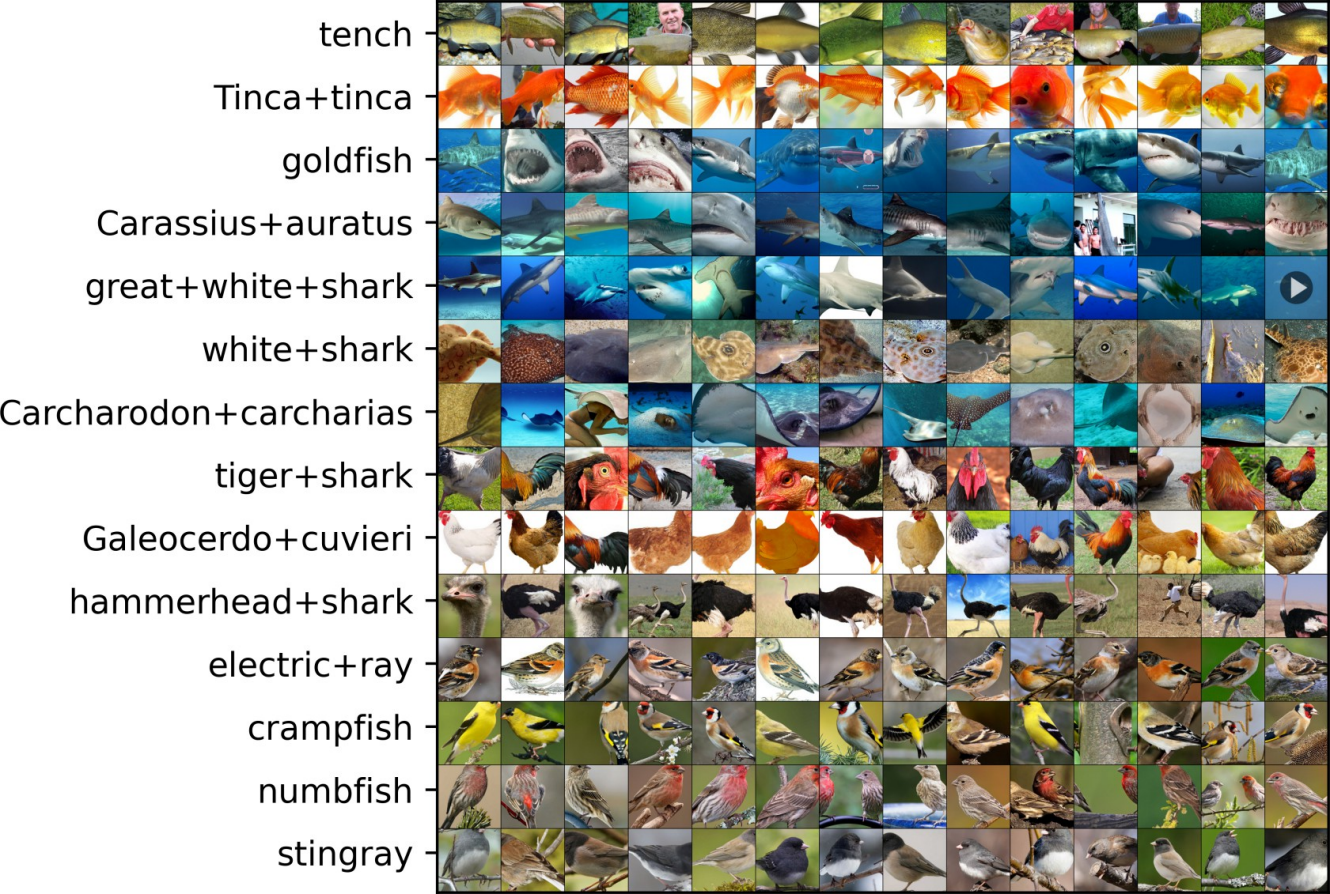
Visualizing samples from WebVision.

*Basic overview*. WebVision [57] is a real-world noisy dataset comprising 2.4 million training images from the internet. Our evaluation of SOS utilizes the first 50 categories from the Google image subset, as done in previous studies. The noise rate is reported about 20% in previous works. Dataset specifics are documented in Table 1 while the visualization of some samples from this set are shown in Fig 8.

*Experimental setup*. The hyperparameters of this set are detailed in Table 2. The learning rate is linearly reduced after warm-up, and *λ_u_* = 0. The “w_da” mirrors that of CIFAR-10/100, while the “s_da” adopts the ImageNet-Policy.

#### The details of Clothing1M

**Fig 9:**
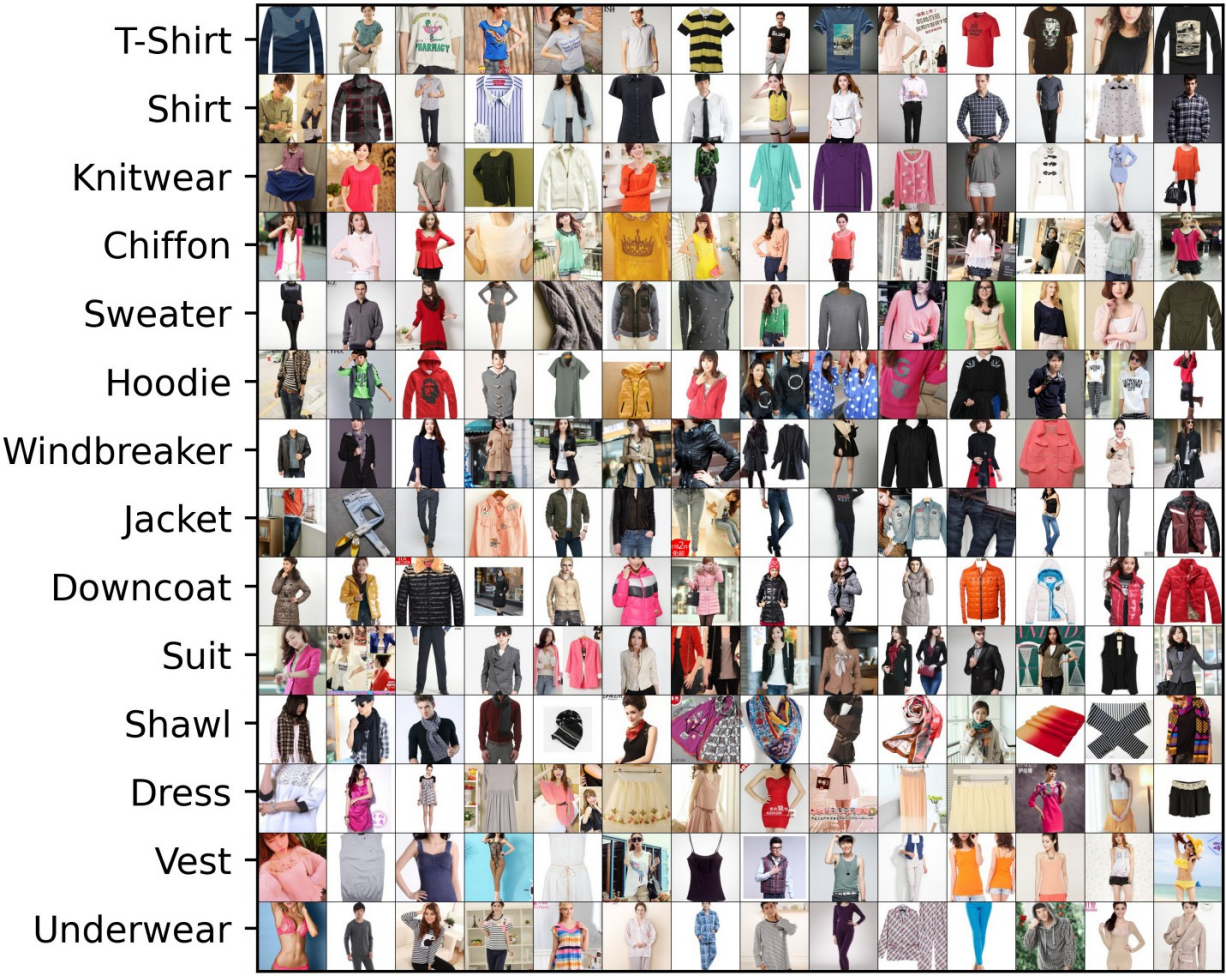
Visualizing samples from Clothing1M. We randomly select 10 images from each of the first 10 categories for display from CIFAR datasets and 14 images from each of the first 14 categories from WebVision and Clothing1M.

*Basic overview*. Clothing1M [58] is a real-world noisy dataset with 14 classes of training data. This dataset is crawled from several shopping sites and contains 38.5% noisy labels in the training set. Its specifics are outlined in Table 1 and visualization of samples are shown in Fig 9.

*Experimental setup*. The hyperparameters used in this study are detailed in Table 2. Notably, we exclusively utilize the 1M training images for training, without employing an extra clean validation set. We randomly sample 32K instances from the entire dataset to implement SOS at each epoch, following [34, 35, 37, 41]. The learning rate is linearly reduced after warm-up, and *λ_u_* = 0. The data augmentation strategy employed is identical to that used in WebVision.

### Experimental results on synthetic datasets

We implement the SOS framework on two synthetic noisy datasets, examining a range of noise rates and types. For the synthetic label noise, we present the results for symmetric noise at {20%, 50%, 80%, 90%} and asymmetric noise at {10%, 30%, 40%}, in line with the methodologies of previous studies.

#### Results on CIFAR-10

**Fig 10:**
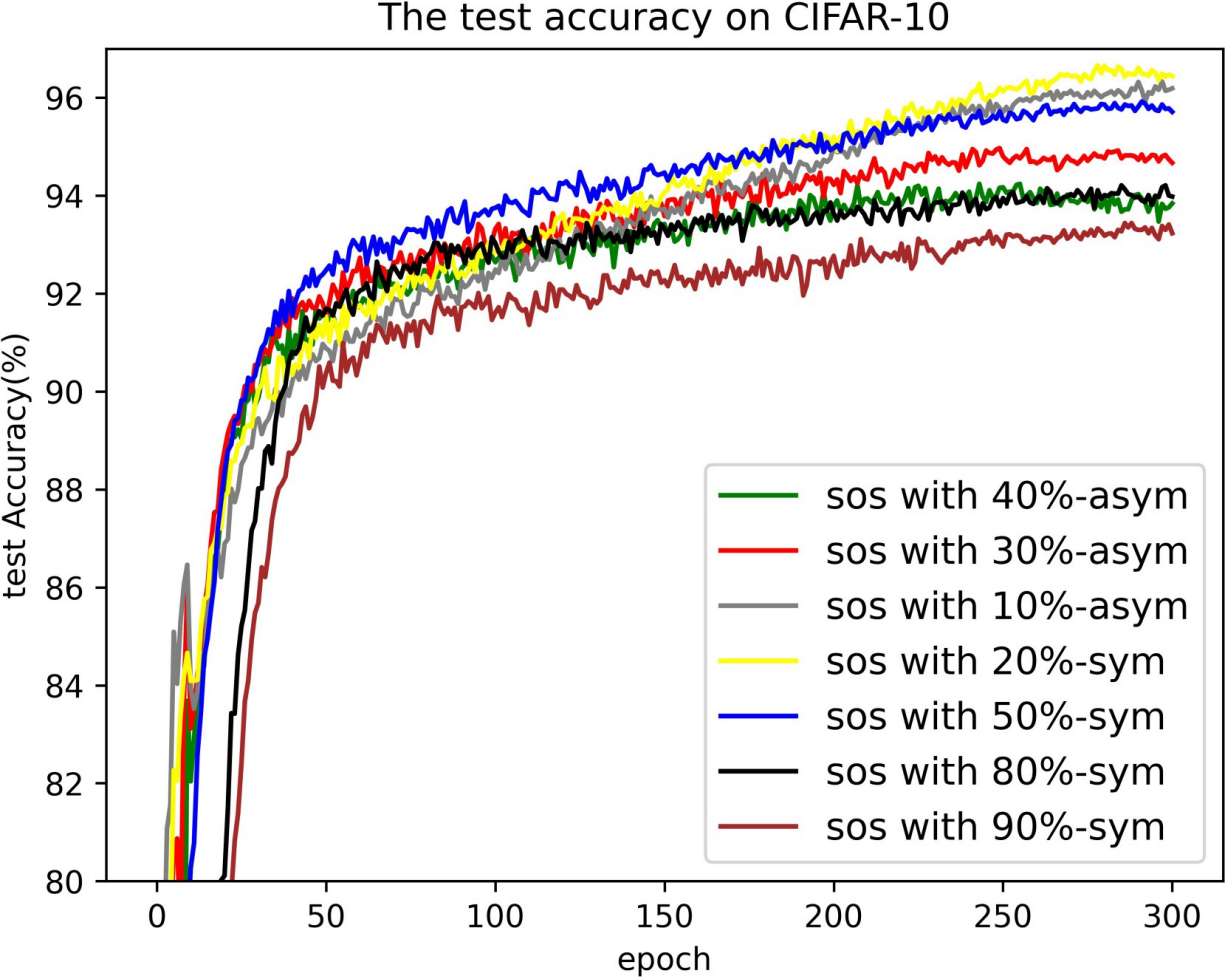
Curve of test accuracy on CIFAR-10. The test results of SOS on CIFAR-10 with different noise rates.

**Fig 11:**
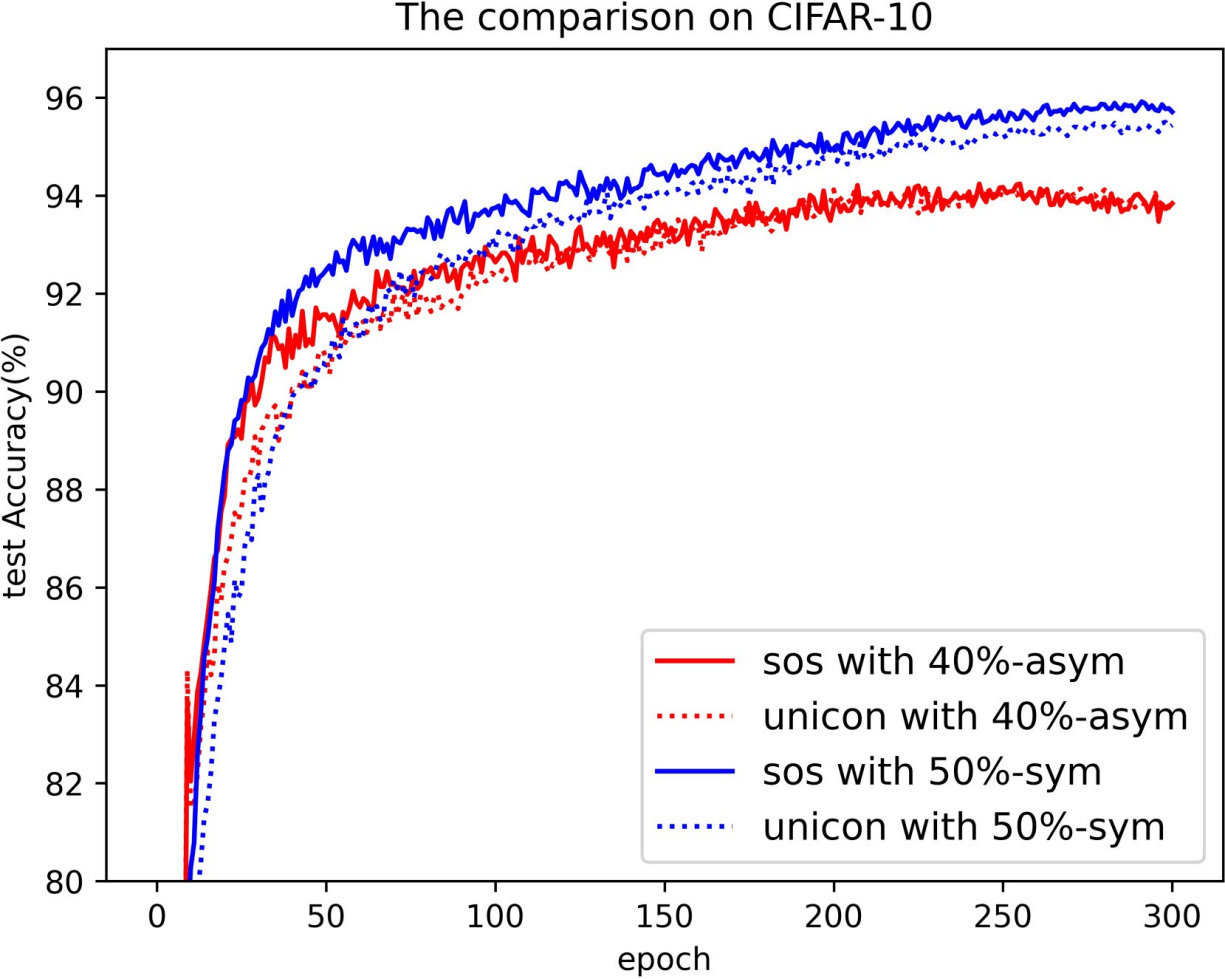
The comparison of classification performance between SOS and UNICON. The steep drop in two figures means the end of the warm-up stage.

**Fig 12:**
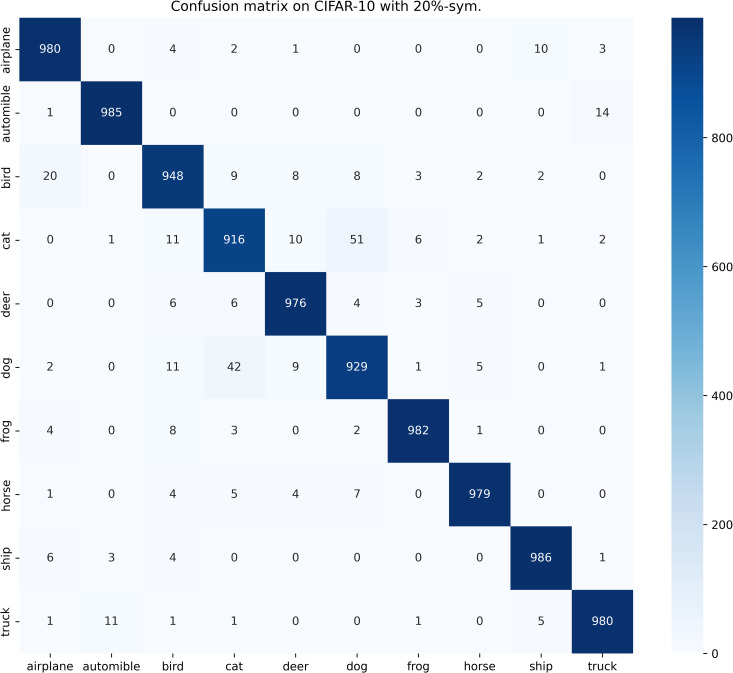
Confusion matrix of SOS on the test set of CIFAR-10 when trained with 20%-sym. scenario.

**Fig 13:**
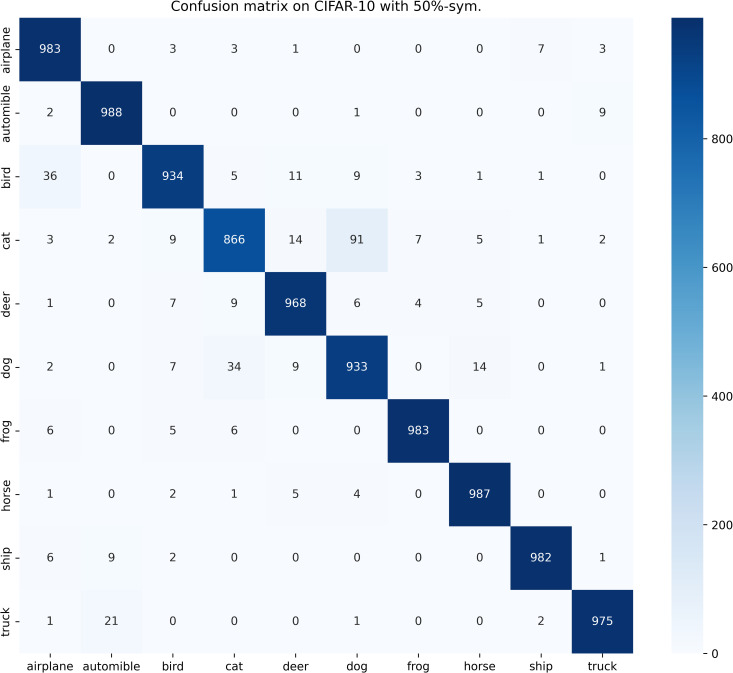
Confusion matrix of SOS on the test set of CIFAR-10 when trained with 50%-sym. scenario.

**Fig 14:**
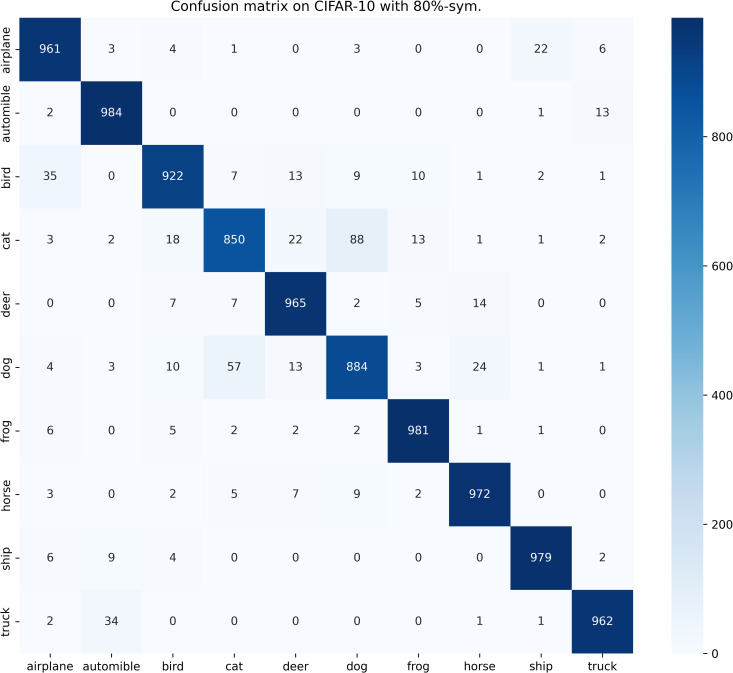
Confusion matrix of SOS on the test set of CIFAR-10 when trained with 80%-sym. scenario.

**Fig 15:**
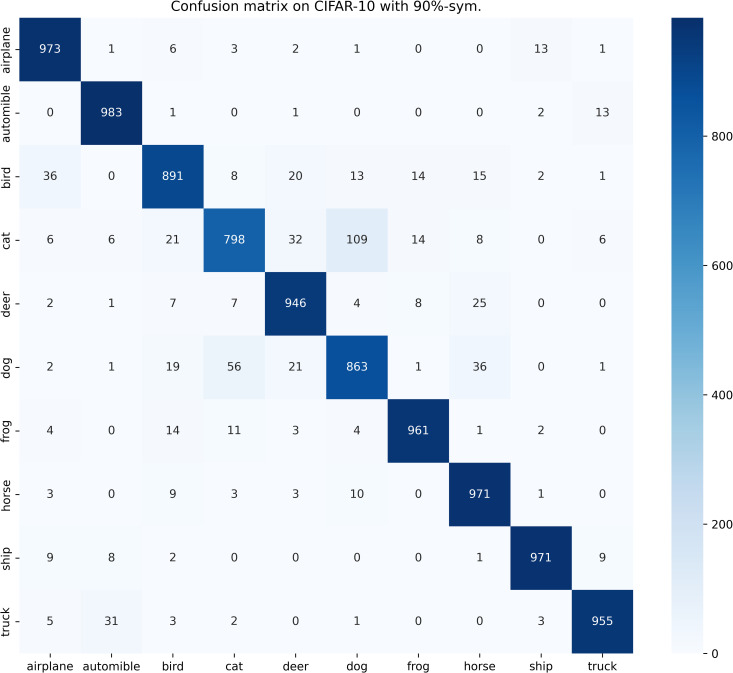
Confusion matrix of SOS on the test set of CIFAR-10 when trained with 90%-sym. scenario.

**Fig 16:**
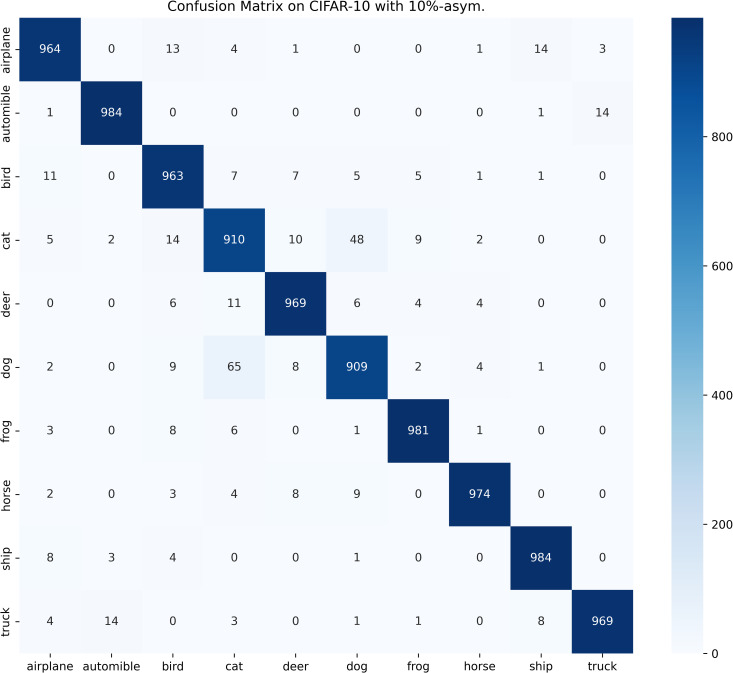
Confusion matrix of SOS on the test set of CIFAR-10 when trained with 10%-asym. scenario.

**Fig 17:**
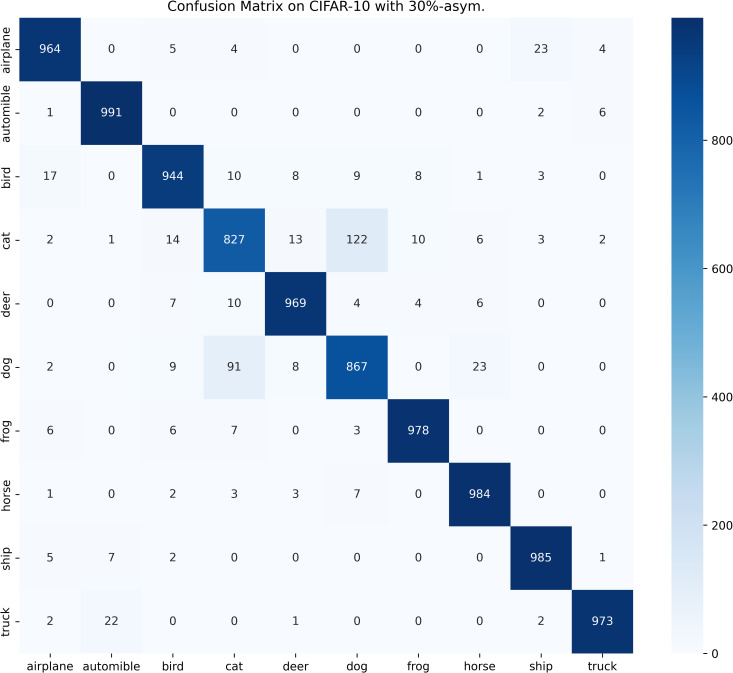
Confusion matrix of SOS on the test set of CIFAR-10 when trained with 30%-asym. scenario.

**Fig 18:**
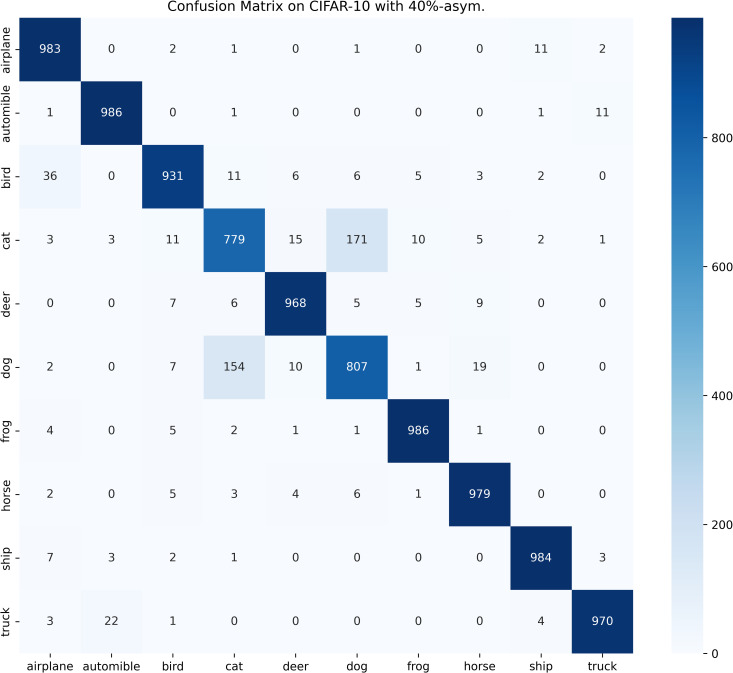
Confusion matrix of SOS on the test set of CIFAR-10 when trained with 40%-asym. scenario.

Table 3 compares the performance of SOS with other SOTA benchmarks on the CIFAR-10 dataset across two types of noise. Although most methods report only the best test accuracies for this dataset, we present both the highest accuracies achieved during the entire training process and the average test accuracies over the last 10 epochs. These results are provided for various noise rates and types. Notably, SOS surpasses nearly all other SOTA baselines. The only exceptions are under the 40%-asym noise condition, where SOS is marginally outperformed by DISC and LongReMix by 0.4% and 0.5%, respectively. This slight underperformance is attributed to these two methods adapting their hyperparameters for different noise rates, whereas SOS employs consistent hyperparameters across conditions. However, in experiments with 50%/80%-sym noise, SOS significantly leads by 0.8%/7% and 0.8%/1.4%, respectively, over these methods. This demonstrates SOS’s ability to maintain robustness and effectiveness across varying noise types and ratios. Furthermore, as detailed in the Proposed SOS framework section, when the noise ratio is excessively high (e.g., 50%-sym), the size of the dataset obtained by existing sample selection methods is considerably smaller than the unlabeled set. Consequently, this discrepancy hinders DNNs from fully learning the representations in the unlabeled samples when employing robust training with CL and SSL techniques, thus limiting performance enhancement. Addressing this issue, our study introduces an SOS that encourages models to extract more representation from the unlabeled dataset, leading to substantial improvements even in contexts of high label noise. For instance, under the 90%-sym noise condition, SOS outperforms co-teaching+, DivideMix, MOIT+, Sel-CL+, UNICON, LongReMix, and TCL by 45%, 16%, 17.3%, 10.1%, 12%, and 1.2%, respectively. Even in asymmetric noise scenarios, SOS maintains superiority over nearly all methods except in the 40%-asym case. Fig 10 illustrates the test accuracy of SOS on CIFAR-10. In addition, Fig 11 compares the classification performance of SOS and UNICON under 50%-sym and 40%-asym conditions, showing that SOS not only converges faster than UNICON but also achieves superior performance. In addition, we provide other common metrics for our method on CIFAR-10, such as precision, recall, F1-score, and confusion matrix. The first three metrics are shown in Table 3, while the confusion matrix obtained on the test set at the final epoch is illustrated in Figs 12–18. By observing the results of accuracy, precision, recall, and F1-score in Table 3, we can see that the four metrics are very close to each other. Since most existing LNL methods only report accuracy, we follow this practice for fair comparison in the subsequent experiments.

**Table 3:** Results on CIFAR-10 using PreAct ResNet-18.

Noise rates		Test accuracy (%) on CIFAR-10						
		Sym.				Asym.		
		20%	50%	80%	90%	10%	30%	40%
Standard CE		86.8	79.4	62.9	42.7	88.8	81.7	76.1
Co-teaching [29]		86.5	76.1	25.4	-	87.2	84.7	75.7
Co-teaching+ [60]		89.5	85.7	67.4	47.9	-	-	-
Mixup [53]		95.6	87.1	71.6	52.2	93.3	83.3	77.7
GCE [25]		86.6	81.9	54.6	21.2	89.5	80.6	76.0
PENCIL [23]		92.4	89.1	77.5	58.2	93.1	92.6	91.6
M-correction [12]		93.8	91.9	86.6	68.7	89.6	92.2	91.2
DivideMix [34]		95.7/96.1	94.4/94.6	92.9/93.2	75.4/76.0	-	-	92.1/93.4
ELR+ [62]		95.8	94.8	93.3	78.7	95.4	94.7	93.0
GCE+CL [61]		90.0	89.3	73.9	36.5	91.1	82.2	78.1
MOIT+ [31]		94.1	91.8	81.1	74.7	94.2	94.3	93.3
Sel-CL+ [37]		95.5	93.9	89.2	81.9	95.6	94.5	93.4
UNICON [35]		96.0	95.6	93.9	90.8	95.3	94.8	94.1
LongReMix [41]		96.0/96.3	94.8/95.1	93.3/93.8	79.1/79.9	-	-	**94.3/94.7**
DISC [40]		96.1	95.1	84.7	-	-	-	**94.6**
TCL [39]		95.0	93.9	92.5	-	-	-	92.6
SOS	Accuracy	**96.5/96.7**	**95.8/95.9**	**94.0/94.2**	**93.3/93.4**	**96.1/96.3**	**94.8/95.0**	93.8/94.2
	Precision	**96.4/96.6**	**96.0/96.2**	**94.4/94.7**	**93.1/93.3**	**96.2/96.3**	**94.9/95.1**	93.8/94.2
	Recall	**96.4/96.6**	**96.0/96.2**	**94.4/94.7**	**93.2/93.3**	**96.1/96.3**	**94.9/95.1**	93.8/94.2
	F1-score	**96.4/96.6**	**96.0/96.2**	**94.4/94.7**	**93.1/93.3**	**96.1/96.3**	**94.9/95.1**	93.8/94.2

We report the “last/best” for SOS while only “best” are shown in most previous works. The best results are in bold.

**Fig 19:**
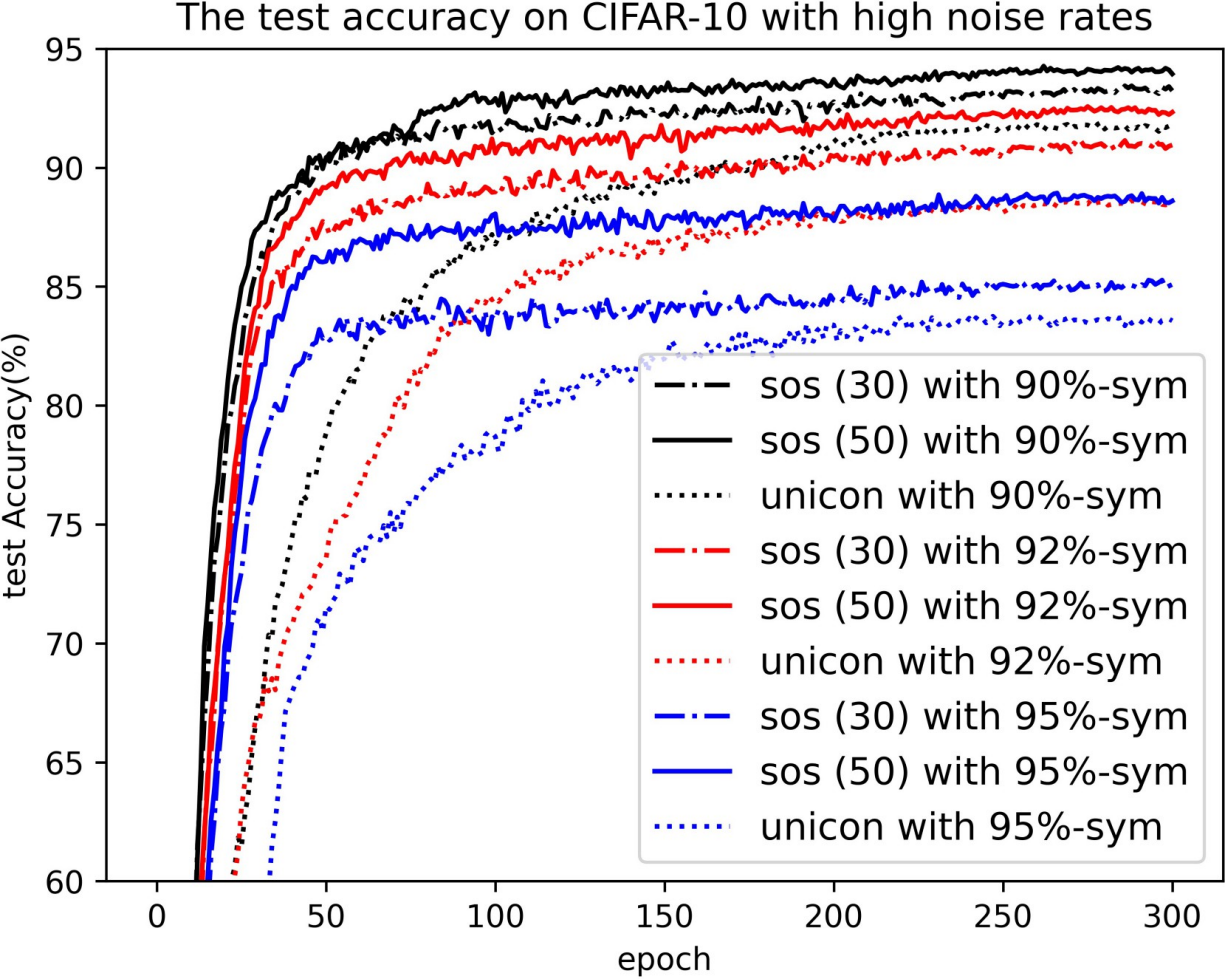
Test curve of SOS and UNICON on CIFAR-10 with severe label noise. “SOS (30)” and “SOS (50)” represent the results of our method when *λ_u_* = 30 and *λ_u_* = 50, respectively.

Moreover, Table 4 presents a comparison of the test results between our proposed method and other SOTA methods, such as those mentioned in [35], under severe symmetric label noise conditions {specifically 90%, 92%, and 95%}, posing a massive challenge for previous methodologies. Impressively, our method demonstrates satisfactory performance even in these demanding scenarios. Notably, SOS significantly outperforms UNICON by 1.5%, 2.5%, and 1.5% in experiments conducted with 90%, 92%, and 95% symmetric label noise, respectively. Furthermore, the efficacy of our method is visually represented through the test accuracy curve shown in Fig 19, providing a more intuitive understanding of its performance. In the scenario of 95% symmetric noise, taking the bird category as an example, current research on symmetric noise mostly involves randomly flipping the labels of 95% of bird samples to any other category (including the bird category) during practical operations [34, 35, 36, 51]. This means a certain proportion of samples still retain the bird category label. Therefore, in reality, the number of clean label samples for the bird category should be greater than 250 (i.e., 5%×5000), approaching 725 (i.e., 5%×5000+95%×5000/10). The distribution of samples for other categories is similar. From a statistical perspective, there is still a possibility of correct classification in this scenario. Unfortunately, DivideMix’s performance shows drastic deterioration, while Unicon and our method maintain stable clustering capabilities by introducing balanced selection and contrastive loss strategies for feature extractors. Combining with partitioned clean label samples can ensure the robustness of the classifier. Additionally, since SOS introduces oversampling techniques, it can assist the Contrastive Learning module in more thoroughly mining the information carried by unlabeled samples, thereby achieving better performance in high noise ratio scenarios compared with Unicon.

**Table 4:** Classification performance on CIFAR-10 with heavy symmetric noise.

Noise rates	Test accuracy (%) on CIFAR-10		
	90%-Sym	92%-Sym	95%-Sym
DivideMix [34]	76.08	57.62	51.28
UNICON [35]	90.81	87.61	80.82
UNICON [35]*	91.67/91.91	88.51/88.64	83.57/83.79
SOS (*λ_u_* = 30)	**93.28/93.44**	**90.96/91.16**	**85.12/85.27**
SOS (*λ_u_* = 50)	**93.07/94.09**	**92.34/92.57**	**88.65/88.97**

The last/best results of our method are shown.

The “*” denotes the reproduction results.

#### Results on CIFAR-100

**Fig 20:**
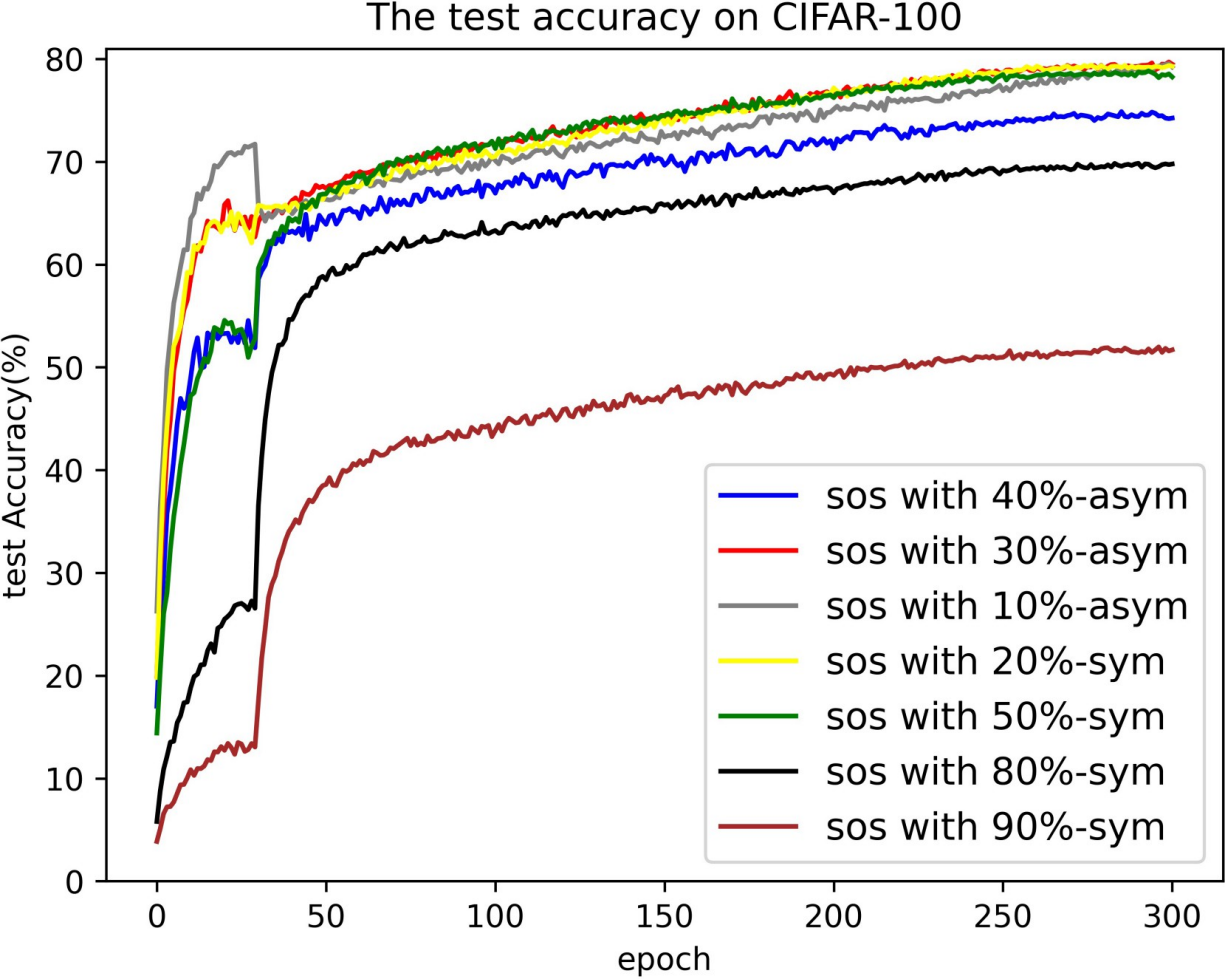
Curve of test accuracy on CIFAR-100.

**Fig 21:**
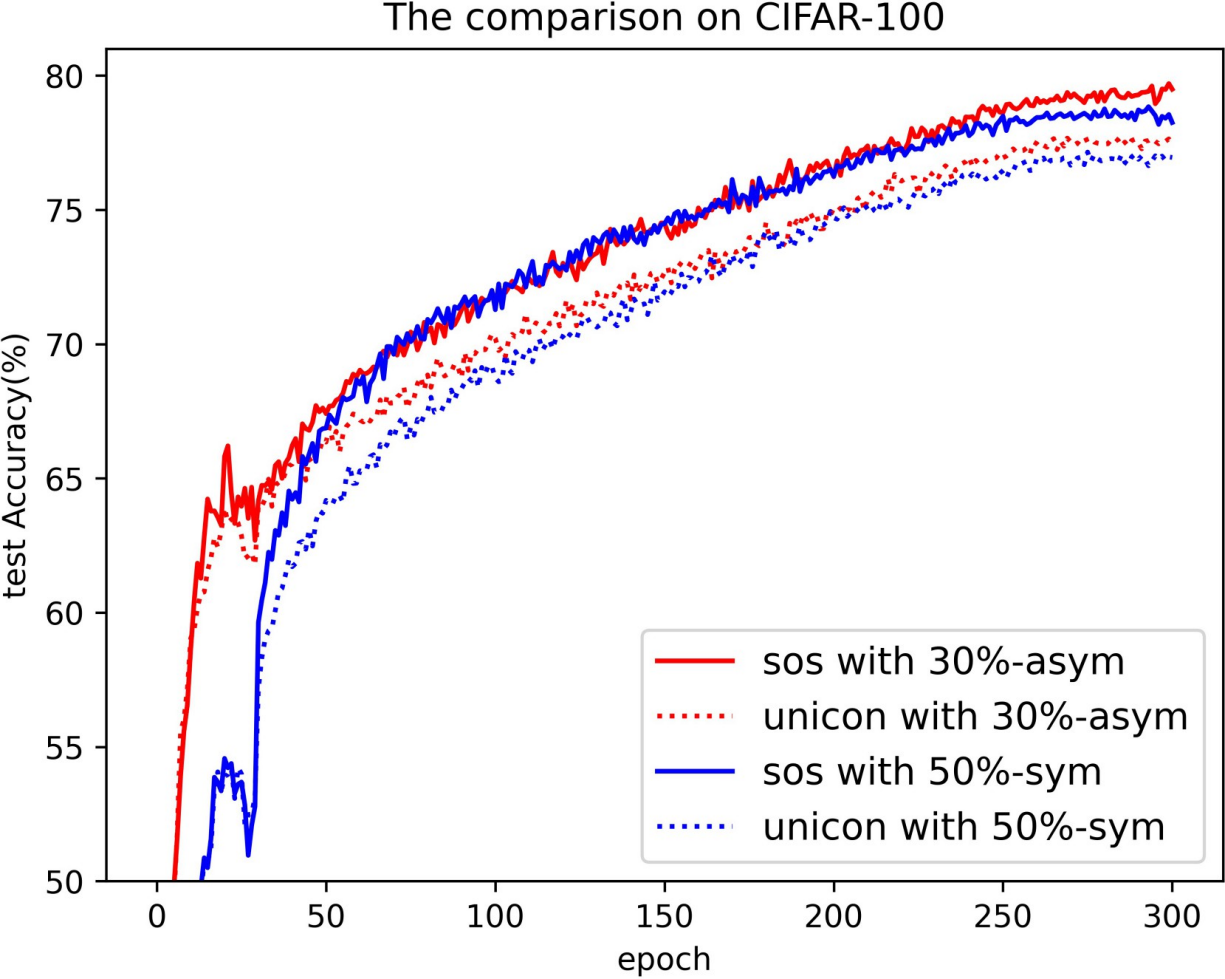
The comparison between SOS and UNICON.

Table 5 presents the highest test accuracy achieved on CIFAR-100 throughout the entire training process, as well as the average test accuracies over the last ten epochs. In these comparisons, SOS consistently outperforms nearly all other SOTA methods. Notably, even when the noise rate is relatively low—a scenario where current methods match the accuracy of clean datasets—SOS still maintains a significant lead. More impressively, SOS’s superiority is evident in severe noise environments, such as 80%-sym. For instance, under the 80%-sym condition, SOS surpasses co-teaching, DivideMix, ELR+, UNICON, DISC, LongReMix, and TCL by 65%, 10%, 9%, 6%, 12%, 7%, and 5%, respectively. This indicates that SOS can approximate a global minimizer comparable with clean datasets. However, it is important to acknowledge that when dealing with higher noise rates and larger categories, the SOS method falls slightly behind the current SOTA methods. This limitation stems from the uniform selection approach and the consistent use of the same hyperparameters during training. Fortunately, datasets with such characteristics are rare in practice. In addition, no single method consistently outperforms SOS, highlighting the stability of our approach. For a more intuitive comparison, the test accuracy curve of SOS on CIFAR-100 is provided in Fig 20. Fig 21 compares SOS and UNICON under 50%-sym and 30%-asym conditions, clearly demonstrating SOS’s superiority over current SOTA methods.

**Table 5:** Results on CIFAR-100.

Noise rates	Test accuracy (%) on CIFAR-100						
	Sym.				Asym.		
	20%	50%	80%	90%	10%	30%	40%
Standard CE	62.0	46.7	19.9	10.1	68.1	53.5	44.5
Co-teaching [29]	49.2	35.1	5.7	-	54.1	49.6	43.7
Co-teaching+ [60]	65.6	51.8	27.9	13.7	-	-	-
Mixup [53]	67.8	57.3	30.8	14.6	72.4	57.6	48.1
GCE [25]	59.2	47.8	15.8	7.2	68.0	51.4	42.9
PENCIL [23]	68.1	56.4	20.7	8.8	76.1	59.3	48.3
M-correction [12]	73.4	65.4	47.6	20.5	67.1	58.6	47.4
DivideMix [34] *	76.9/77.3	74.2/74.6	59.6/60.2	31.0/31.5	69.5	68.3	51.0
ELR+ [62]	77.6	73.6	60.8	33.4	77.4	75.1	74.0
GCE+CL [61]	68.1	53.3	22.1	8.9	70.2	52.6	44.1
MOIT+ [31]	75.9	70.6	47.6	41.8	77.4	75.1	74.0
Sel-CL+ [37]	76.5	72.4	59.6	48.8	78.7	76.4	74.2
UNICON [35]	78.9	77.6	63.9	44.8	78.2	75.6	74.8
DISC [40]	78.8	75.2	57.6	-	-	-	**76.5**
TCL [39]	78.0	73.3	65.0	**54.5**	-	-	-
LongReMix [41]	77.5/77.9	74.9/75.5	61.7/62.3	30.7/34.7	-	-	54.9/59.8
SOS (Ours)	**79.2/79.4**	**78.5/78.8**	**69.6/69.9**	51.6/52.0	**79.2/79.6**	**79.4/79.7**	74.5/74.9

The best results are in bold.

The “*” denotes the results are from [37] and [34].

### Experimental results on real-world datasets

We applied the SOS method to three real-world noisy datasets. Specifically, for the CIFAR-N dataset, which contains real-world noise, we comprehensively compared SOS’s performance against the current SOTA methods across various noise types.

#### Results on CIFAR-N

**Fig 22:**
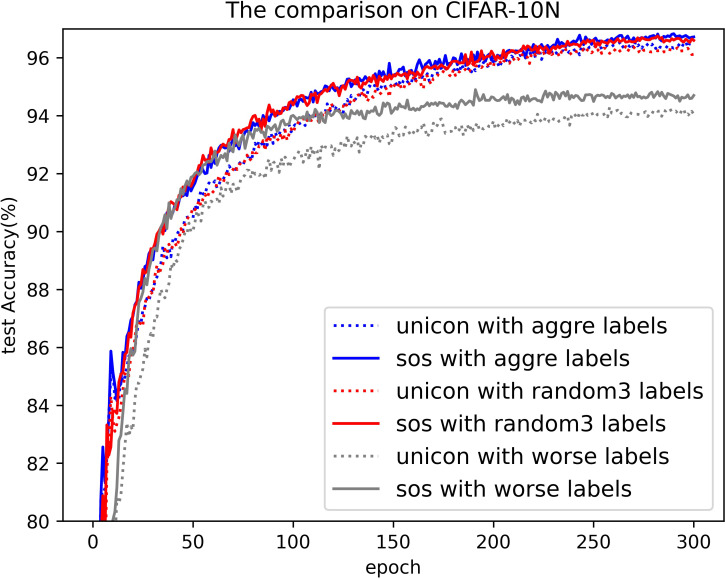
Comparison of test accuracy on CIFAR-10N.

**Fig 23:**
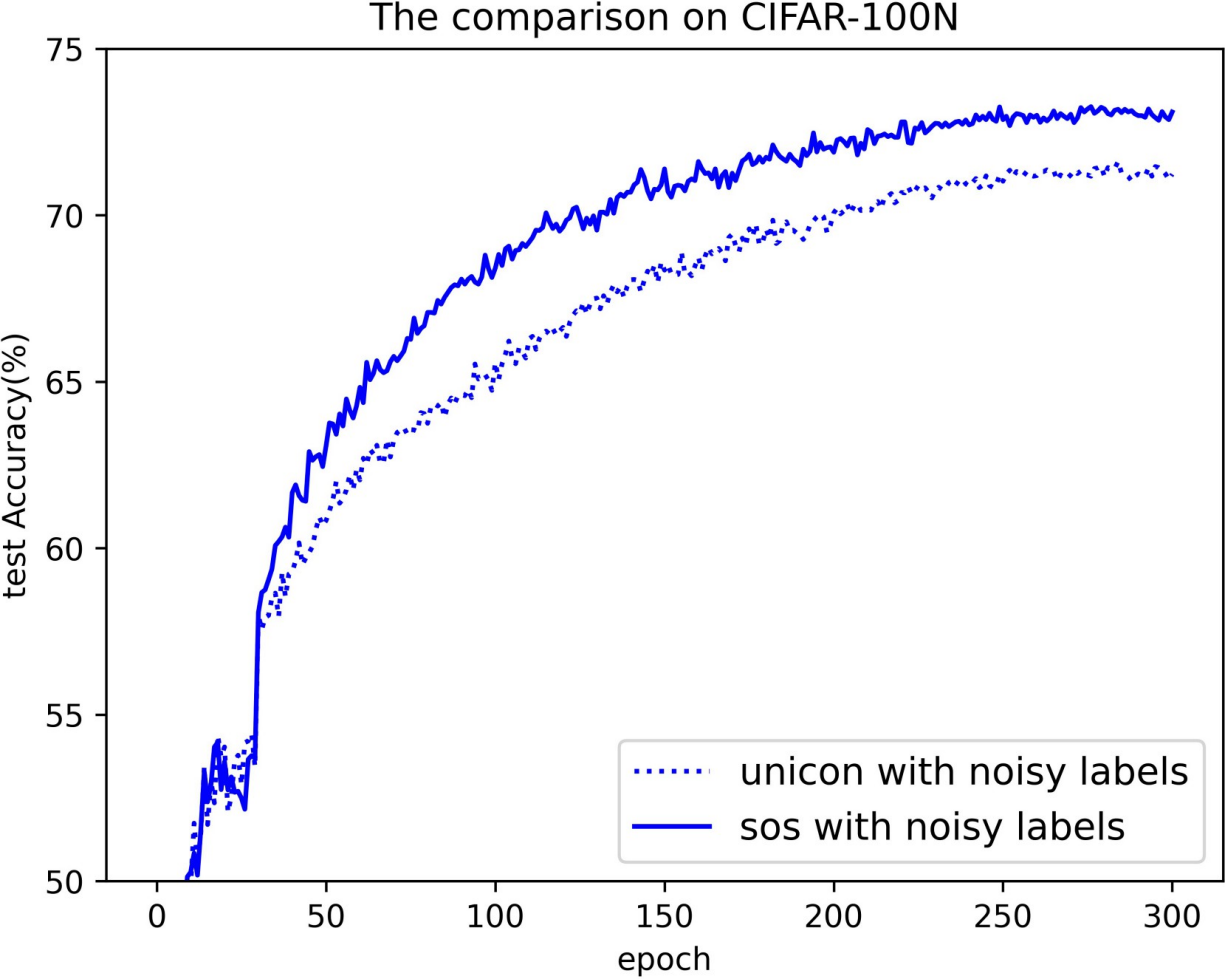
The comparison on CIFAR-100N.

Table 6 presents the results obtained using SOS on the CIFAR-N dataset, with Figs 22 and 23 illustrating a comparative analysis between SOS and UNICON on this dataset. CIFAR-N, a real-world noisy dataset compiled via crowdsourcing platforms, offers a more realistic assessment of the methods’ effectiveness and robustness. Unlike most methods that report only the best test accuracies on this dataset, we provide both the highest accuracies throughout the training process and the average test accuracies over the final 10 epochs, encompassing various noise rates and types. In addition, we have included results for UNICON obtained by running its publicly available code. Consistently, SOS outperforms almost all other SOTA baselines, particularly under severe label noise scenarios, such as “worst” and “noisy.” It is noteworthy that many SOTA methods adjust their hyperparameter settings for the seven noise types, whereas we maintained almost identical settings to those used for CIFAR-10/100. Despite this, our method demonstrates superior performance. For instance, under the “worst” label noise condition (CIFAR-10N), SOS surpasses co-teaching, DivideMix, ELR+, JoCoR+, UNICON, ILL, and PLS by 11%, 2.4%, 3.8%, 0.6%, 1.4%, and 1.2%, respectively. In the more challenging “noisy” scenario on CIFAR-100N, SOS still leads these methods by margins of 13%, 2.1%, 14%, 1.6%, 5.2%, and 0.1%. These results across various experiments on this dataset demonstrate that SOS, with its robust representation learning from unlabeled samples, consistently outperforms other SOTA methods. Furthermore, as shown in Figs 22 and 23, SOS converges faster and achieves higher accuracy than current SOTA methods.

**Table 6:** Results on CIFAR-N.

Methods	Test accuracy (%) on CIFAR-N						
	Clean	Aggregate	Random1	Random2	Random3	Worst	Noisy
Standard CE	92.92	87.77	85.02	86.46	85.16	77.69	55.50
Co-teaching [29]	93.35	91.20	90.33	90.30	90.15	83.83	60.37
Co-teaching+ [60]	92.41	90.61	89.70	89.47	89.54	83.26	57.88
GCE [25]	92.83	87.85	87.61	87.70	87.58	80.66	56.73
Peer Loss [63]	93.99	90.75	89.06	88.76	88.57	82.00	57.59
ELR [62]	93.45	92.38	91.46	91.61	91.41	83.58	58.94
ELR+ [62]	95.39	94.83	94.43	94.20	94.34	91.09	66.72
DivideMix [34]	95.37	95.01	95.16	95.23	95.21	92.56	71.13
JoCoR [33]	93.40	91.44	90.30	90.21	90.11	83.37	59.97
VolMinNet [64]	92.14	89.70	88.30	88.27	88.19	80.53	57.80
UNICON [35]*	96.2/96.3	96.5/96.6	96.4/96.5	96.2/96.3	96.2/96.4	94.1/94.3	71.3/71.6
SOP [67]	N/A	95.61	95.28	95.31	95.39	93.24	67.81
ILL [65]	-	96.40	96.06	95.98	96.10	93.55	68.07
PLS [66]	-	96.09	95.86	95.96	96.10	93.78	73.25
SOS (Ours)	**96.4/96.5**	**96.7/96.8**	**96.6/96.7**	**96.4/96.6**	**96.6/96.8**	**94.6/94.9**	**73.0/73.3**

The best results are in bold. The results are from [56].

The “*” implies we run the algorithm by the public code.

#### Results on WebVision

Table 7 details the highest test accuracy achieved on WebVision throughout the training process. In line with prior research, we report the test accuracies for the validation set in WebVision and the ILSVRC2012 validation set. In addition, we include both top-1 and top-5 accuracies for these two validation sets. Although SOS is somewhat less effective than LongReMix on WebVision, it is important to note that LongReMix utilizes a two-stage sample selection method, which requires twice the training time of SOS. This difference in training time becomes more significant in large-scale datasets. Although TCL achieved the highest performance on WebVision, surpassing our method by 1.1%, our method outperformed TCL on ILSVRC12 with a margin of 1.44%. Thus, overall, the method presented in this study demonstrates greater stability and consistently better performance across both validation sets, which is a significant advantage in practical applications.

**Table 7:** Results on WebVision using pre-trained ResNet-50.

Methods	WebVision		ILSVRC12	
	Top-1 (%)	Top-5 (%)	Top-1 (%)	Top-5 (%)
Co-teaching [29]	63.58	85.20	61.48	84.70
DivideMix [34]	77.32	91.64	75.20	90.84
ELR [62]	76.26	91.26	68.71	87.84
ELR+ [62]	77.78	91.68	70.29	89.76
RRL [68]	77.8	91.3	74.4	90.9
MOIT [31]	77.9	91.9	73.8	91.7
TCL [39]	**79.1**	92.3	75.4	92.4
UNICON [35]	77.60	**93.44**	75.29	**93.72**
LongReMix [41]	78.92	**92.32**	-	-
SOS (Ours)	78.00	92.04	**76.84**	**92.76**

The best and next best accuracies are in bold.

#### Results on Clothing1M

Table 8 displays the highest test accuracy achieved on the Clothing1M dataset over all training epochs. Although TCL and UNICON show a slight performance edge over SOS, it is important to consider the differences in the running environments and the substantial scale of Clothing1M, which comprises one million images. Given these factors, SOS, TCL, and UNICON exhibit comparable performance on this dataset. We replicated UNICON using its publicly available code and maintained the same hyperparameter settings as those used in our method. The results presented in Table 8 are 1.0% lower than UNICON’s originally reported results, suggesting that these three methods are essentially on par in terms of performance. Furthermore, SOS demonstrates competitiveness with, or superiority over, other LNL methods from 2023, such as LongReMix, OT-Filter, DISC, and TCL.

**Table 8:** Results on Clothing1M using pre-trained ResNet-50.

Methods	Test accuracy (%)
Standard CE	68.94
Co-teaching [29]	71.70
Co-teaching+ [60]*	59.32
JoCoR [33]	70.30
DivideMix [34]	74.46
ELR+ [62]	74.39
TCL [39]	74.80
M-correction [12]	71.00
SCE [28]	71.02
PENCIL [23]	73.49
UNICON [35]	**74.98**
UNICON [35]†	73.98
TCC-Net [32]*	70.46
DISC [40]	73.72
OT-Filter [36]	74.00
LongReMix [41]	74.38
SOS (Ours)	**74.50/74.72**

The “*” indicates the model is trained with PreAct ResNet-18.

“†” denotes the reproduction results. The best accuracy is in bold. The last/best results of SOS are given.

### Ablation results

As shown in Tables 2–4, we utilize nearly identical hyperparameter settings for different noise scenarios on the CIFAR datasets. However, existing LNL methods such as DivideMix, LongReMix, and DISC (which employ the same SSL technique as our method) typically require real-time adjustments of multiple hyperparameters, including thresholds and loss weights, based on noise type and noise ratio. In contrast, our method utilizes nearly the same hyperparameter settings across various noise scenarios for this dataset, which sufficiently demonstrates the robustness of our approach. Additionally, our method is inspired by UNICON, which has already shown that DNNs are insensitive to the hyperparameters listed in Table 2 when using uniform selection approach and SSL technique. Therefore, sensitivity analysis of hyperparameters to noise information is not repeated here, please refer to Section 4.3 of [34] and Section 11 of [35] for further analyses. Moreover, SOS consistently applies the same hyperparameter settings across all datasets, except for *λ_u_*, and the effects of *λ_u_* have been analyzed in previous works [34, 35, 41], revealing that SOS exhibits relative insensitivity to variations in hyperparameter settings. Consequently, our analysis focuses primarily on the effects of the SOS, comparing scenarios with (w/) and without (w/o) this strategy on test accuracy, specifically using the CIFAR-10 and CIFAR-N datasets. Table 9 presents the results of the ablation study, highlighting only the best outcomes observed throughout all epochs. The results indicate that the SOS enhances model performance by leveraging the feature representations in label-free samples more effectively. This finding supports the utility of noisy samples in improving the classification performance and robustness of DNNs.

**Table 9:** Ablation results of SOS.

**Noise rates**	**Test accuracy (%) on CIFAR-10**						
	**Sym.**				**Asym.**		
	**20%**	**50%**	**80%**	**90%**	**10%**	**30%**	**40%**
w/o. OS	96.0	95.6	93.9	90.8	95.3	94.8	94.1
w. OS	**96.7**	**95.9**	**94.2**	**92.0**	**96.3**	**95.0**	**94.2**
**Noise types**	**Test accuracy (%) on CIFAR-N**						
	**Clean**	**Agg** ^**a**^	**Rand1**	**Rand2**	**Rand3**	**Worst**	**Noisy**
w/o. OS	96.3	96.6	96.5	96.3	96.4	94.3	71.6
w. OS	**96.5**	**96.8**	**96.7**	**96.6**	**96.8**	**94.9**	**73.3**

The best results are in bold. “w/o” implies without the oversampling strategy and “w” implies using the oversampling strategy. “OS” represents oversampling strategy.

^a^ “Agg” represents aggregate.

## Discussion

### Efficiency

Table 10 shows a comparison of training times between our method and UNICON, and DivideMix in CIFAR-10 scenarios with 50% symmetric noise and 40% asymmetric noise. All experiments are conducted on a server equipped with a single 4090 GPU. We set the “num_worker” of the dataloader to 0 in all experiments. As clearly indicated in the table, although the introduction of the oversampling technique adds some training time, the overhead is not significant. Since we only need to train once, the testing phase time is identical, and we achieve better performance. Therefore, we consider the additional overhead to be acceptable.

**Table 10:** The training time cost (hours, i.e., “h”) on CIFAR-10 with 50% symmetric noise.

Noise scenario	Standard CE	DivideMix	UNICON	Ours
CIFAR-10 with 50%-sym.	1.5h	9.9h	12.6h	19.0h
CIFAR-10 with 40%-asym.	1.5h	13.6h	14.9h	15.8h

All methods are run in windows11 system with a single Nvidia 3090 GPU and a 13900K CPU. “h” represents hours. “sym.” implies symmetric noise and “asym.” implies asymmetric noise.

### Pros

Our extensive experimental comparisons, encompassing both synthetic and real-world noisy datasets, have demonstrated the effectiveness of SOS. Our experiments on CIFAR-10/100 show that SOS offers several advantages over current methods such as DISC, LongReMix, and UNICON, particularly in practical applications. These advantages include robustness to hyperparameter variations, faster convergence, and superior performance under high-noise conditions. The results from three real-world noisy datasets further corroborate these advantages. In addition, we have validated the effectiveness of the SOS through comprehensive ablation studies on both synthetic and real-world datasets. We have established that the label-free samples, identified post-detection, significantly enhance model performance, an aspect previously overlooked in earlier studies.

### Limitation

Despite the aforementioned advantages of our method, limitations are inevitable. Following previous research, we discuss the limitations of our method from the perspectives of robustness [41], generalization [69], and trustworthiness [70]. Firstly, from the experiments on synthetic noise datasets, it can be observed that our method exhibits excellent robustness to most noise scenarios. However, in high-noise scenarios with a large number of classes, its **robustness** still has shortcomings. For example, on the CIFAR-100 with 40% asymmetric noise and 90% symmetric noise, our method significantly lags behind the SOTA methods TCL and DISC, respectively (e.g., 52.0% vs 54.5%, and 74.9% vs 76.5%). This is mainly because, under such extreme noise conditions with many classes, the number of noisy samples is very close to the number of clean samples. For instance, in the 40% asymmetric noise scenario, the apple class has 500 samples, with at least 40% of them flipped to mushrooms. In this case, the number of clean samples in the apple class should be less than 300, and the number of samples labeled as mushrooms should be more than 200. Under these circumstances, our balanced partitioning mechanism faces a significant challenge. However, in CIFAR-10, since the number of samples per class increases to 5000, the difference between clean and noisy samples increases, resulting in better performance. In other words, we can mitigate this issue by increasing the number of samples per class. Additionally, the experimental results on WebVision reveals that, although our method reduces the performance gap on WebVision and ILSVRC12 compared with existing methods such as TCL and LongReMix, a considerable difference remains (i.e., 1.3%), indicating that our method’s **generalization** is insufficient. Finally, in the 40% asymmetric noise scenario of CIFAR-100, the close proximity of noisy and clean samples leads to suboptimal performance of our sample partitioning strategy, indicating poor trustworthiness in this scenarios. Therefore, improving the model’s confidence in noisy samples is a worthwhile focus for future work.

### Adaptability

As illustrated above, one significant advantage of our method is that it achieves better performance while being insensitive to hyperparameters, thus eliminating the need for prior noise information and enhancing its usability. Additionally, as demonstrated by our experiments on several benchmark datasets, our method consistently achieves similar performance across different datasets, indicating its strong generalization capability. Therefore, we believe that our method can be applied to a variety of noise scenarios, e.g., symmetric, asymmetric, and mixed (i.e., real-world [40]) noisy datasets.

## Conclusion

In this study, we propose an advanced SOS designed for LNL. Our research has identified a gap in current SOS methods: their inability to fully harness the potential of representations in label-free samples, which is crucial for boosting model robustness and performance. SOS addresses this issue by integrating an oversampling strategy with SOTA SSL methods. Through comprehensive experiments, we have established that SOS exhibits a low sensitivity to hyperparameter variations and consistently delivers optimal or near-optimal outcomes across a diverse range of datasets.
